# Going around phase defects: Addressing a fundamental flaw of phase singularity detection

**DOI:** 10.1371/journal.pcbi.1013963

**Published:** 2026-10-13

**Authors:** Bjorn Verstraeten, Sebastiaan Lootens, Robin Van Den Abeele, Viktor Van Nieuwenhuize, Arstanbek Okenov, Sander Hendrickx, Arthur Santos Bezerra, Timur Nezlobinskii, Vineesh Kappadan, Balvinder Singh Handa, Fu Siong Ng, Mattias Duytschaever, Nele Vandersickel

**Affiliations:** 1 Department of Physics and Astronomy, Ghent University, Ghent, Belgium; 2 Imperial College London, London, United Kingdom; 3 Department of Cardiology, AZ Sint-Jan Brugge, Bruges, Belgium; Boston University, UNITED STATES OF AMERICA

## Abstract

Phase mapping is a widespread method for identifying rotational electrical activity sustaining cardiac arrhythmias. However, conventional implementations assume that the cardiac phase map is continuous, leading to ill-defined phase indices in regions affected by functional conduction block, fibrosis, or anatomical boundaries. These regions of discontinuous or undefined phase, termed phase defects, lead to both false positive and false negative detections of rotational drivers. This work introduces an improved phase mapping implementation termed *extended phase mapping* that explicitly detects and accounts for phase defects, enabling robust calculation of the phase index around them. Extended phase mapping is applied to (1) simulated excitation patterns using the Fenton–Karma model, (2) experimental optical mapping data of rat ventricular fibrillation, and (3) a clinical CARTO activation map of atrial tachycardia. Across all datasets, the extended approach eliminates erroneous detections and resolves previously missed rotations. Our results demonstrate that proper treatment of phase defects yields a unified and physiologically consistent characterization of all rotational drivers including near-complete and anatomical reentries. Therefore, we propose that explicitly accounting for phase defects, rather than tracking singular points alone, gives a more complete and consistent characterization of rotational dynamics in cardiac tissue.

## Author summary

Detecting rotating electrical activity in the heart is crucial for understanding and treating abnormal heart rhythms. A common method, phase mapping, assigns a timing phase to each region of the heart to identify these rotations. However, in regions affected by scars, blocked conduction, or anatomical boundaries, the phase can become undefined or discontinuous. These so-called phase defects make current methods unreliable, causing false detections or missed rotations. In this study, we introduce an extended method that explicitly identifies phase defects and calculates phase indices around them. We test this approach using computer simulations, experimental recordings from animal hearts, and clinical heart-mapping data. Across all datasets, it eliminates false detections and reveals previously overlooked rotational activity. By properly accounting for phase defects, the extended phase mapping method provides a more reliable and complete characterization of heart rhythms, offering a physiologically meaningful framework for studying electrical dynamics in cardiac tissue.

## Introduction

Cardiac arrhythmias are a major contributor to global mortality. They remain difficult to treat effectively due to patient-specific mechanisms, with rotational electrical activity identified as a key driver [1–3]. Detecting and characterizing such activity has therefore been a central goal in cardiac simulations, experimental, and clinical electrophysiology.

In order to detect rotational activity in optical maps of a rabbit heart, phase mapping was introduced [4]. This method converts the cardiac signal to a phase, after which rotational activity is identified as singular points where the phase cycles through a full rotation of 2π.

Since then, numerous papers have been published that use this approach for analyzing different datasets ranging from simulated [5–8] to experimental [9,10] and clinical [11–16] data. These studies have demonstrated both the promise of using phase mapping for singularity detection and its limitations when confronted with realistic data. The identification of phase singularities using phase mapping can be distorted by several physiological factors, such as functional conduction blocks [7,8,16,17], scar tissue [7,8] and anatomical structures [16]. In particular, functional conduction blocks have been recognized as equally important as phase singularities [18,19] in describing cardiac arrhythmia dynamics. Likewise, anatomical structures play a critical role, especially in the context of ablation therapy of atrial tachycardia [20]. Together, these features underscore the need to reconsider how singularity detection algorithms handle such factors and previous work has already explored deep-learning techniques to overcome these features [21–23].

This paper argues that reliable detection is achievable by using phase mapping, as long as phase defects are explicitly accounted for.

To demonstrate this, two algorithmic approaches are compared: a naive approach that assumes the phase map is continuous except for a finite number of singular points, and an extended approach that explicitly accounts for phase defects [19], defined as regions of discontinuous or undefined phase resulting from collisions with refractory tissue, i.e., functional conduction block, or anatomical and structural obstacles that prevent conduction in certain directions.

Both approaches are applied to three simulated activation patterns to isolate different arising problems, to an experimental optical mapping recording of ventricular fibrillation in a rat heart, and to a clinical activation map of atrial tachycardia to demonstrate the efficacy in a realistic setting. Combined, these results show that naive implementations fail when confronted with discontinuous or unexcitable regions in the phase map, but that reliability can be restored when phase defects are treated properly.

## Materials and methods

Numerous methods exist to detect rotational activity [15,24,25], making it impractical to assess the impact of phase defects on all of them. Therefore, we implemented two representative approaches to phase mapping: a *naive approach*, representative of existing methods in the literature that assume the phase map is continuous except at isolated singularities, and a novel *extended approach* developed in this work, which explicitly accounts for phase defects.

Both approaches are applied to study cases of (1) simulated activation patterns around phase defects to isolate specific problems, (2) an experimental optical mapping recording of ventricular fibrillation in a rat heart to illustrate their impact in a semi-controlled setting, and (3) a clinical CARTO activation map of atrial tachycardia to evaluate the methods in realistic clinical conditions.

### Phase mapping

Phase mapping is, a technique commonly employed to investigate activation dynamics in cardiac tissue [26]. The method involves constructing a phase map that represents the state of the tissue, followed by scanning the entire tissue to compute phase indexes and identify rotational activity. This process naively assumes that the phase map is continuous throughout the tissue, and is therefore referred to as the *naive approach*. In this work, a second method was developed by introducing an additional step before the computation of the phase indices. This step identifies regions where the assumption of a continuous phase field is violated. This extended procedure is referred to as the *extended approach*.

#### Phase.

In cardiology, the state of a cardiac cell changes repeatedly: a cell transitions from a resting state to an excited state and eventually repolarizes back to rest. Due to this repeating behavior, the state of a cardiac cell can be approximated by a phase ϕ(t)∈[−π,π) [26].

In this paper, it is assumed that cardiac cells are activated when their measured voltage crosses a certain threshold or their time-derived voltage is maximal and crosses a threshold. Additionally, we enforce a constant rate of change in the phase between subsequent activations. As such, we construct a phase map ϕ(𝐫,t), which is the collection of phases on the cardiac tissue. We calculate the phase via the sawtooth method based on LATs, illustrated in Fig 1.

**Fig 1:**
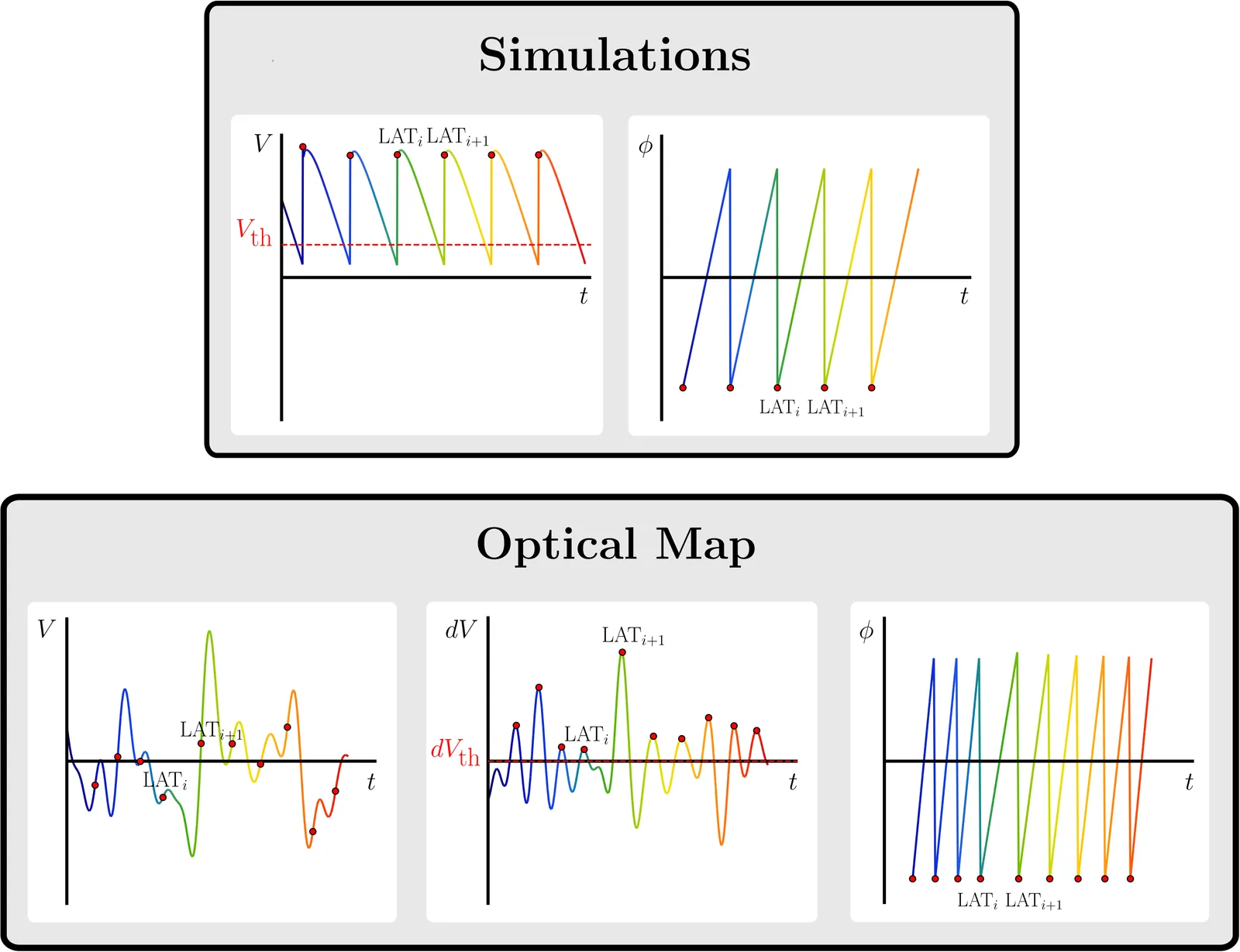
Phase computation in simulated and optical signals. *Simulations:* The first panel illustrates a simulated transmembrane voltage signal along with its annotated LATs calculated using a voltage threshold. The second panel depicts the resulting phases of the simulated voltage signal. *Optical Map:* The first panel shows an optical mapping voltage signal. The second panel illustrates the derivative of the optical mapping signal *dV* along with the annotated LAT values as local maxima of *dV* exceeding a threshold *dV*_th_. The third panel shows the resulting phases of the optical mapping signal.

For the simulations where the signals represent transmembrane voltages, the LATs for each point are annotated by searching for the time step where the normalized voltage *u* crosses the activation threshold for the fast inward current $u_{c}=0.13$ [27]. The optical mapping signals represent unipolar voltages, but contain considerable signal drift. As such, LATs are annotated as the local maxima of the time derivative of the signal exceeding *dV*_th_ = 0 ~ mV [28]. The clinical CARTO data contained pre-calculated LATs, so no conversion was needed. These LATs are then transformed into phases using sawtooth mapping [29], which linearly interpolates the phase between two consecutive LATs. Importantly, points activated fewer than two times are considered inactive and are assigned an undefined phase.

#### Phase index.

The phase index is a topological property defined on a closed curve within a phase map. It quantifies the net change in phase around the curve and can be formally given by:


$$\begin{array}{c} I_{C}=\frac{1}{2\pi }\oint_{C}\nabla \phi (𝐫,t)\cdot d𝐫, \end{array}$$ (1)

where *C* is a closed curve and ϕ(𝐫,t) is the phase along *C*. In contrast to previous formulations that require the phase to be differentiable except at isolated points [5,30], this work only requires the phase to be continuous and piecewise differentiable along the integration path. This allows the phase field to exhibit wavefront collisions, where the derivative of the phase is undefined, while still ensuring that the phase varies continuously along the integration path. Since the phase must return to the same state after a full traversal of the closed curve, the total change in phase around *C* must be an integer multiple of 2π. Consequently, the phase index $I_{C}$ can only take integer values.

In electrophysiology, the phase index is used to identify certain activation patterns. Functional reentry, for instance, is typically associated with small curves that exhibit a non-zero phase index, indicating the presence of a phase singularity [4]. Similarly, anatomical reentry can be characterized by evaluating curves that encircle anatomical structures, and calculating whether their phase index is non-zero [31].

However, in cardiac tissue, the assumption of the phase map being continuous is not always valid. Regions may exist where ϕ(𝐫,t) exhibits discontinuities, rendering the phase index ill-defined for curves that intersect them. Such regions are referred to as *phase defects* [19], which will be discussed in the following section.

#### Naive implementation of phase mapping analysis.

After constructing the phase map ϕ(𝐫,t), the cardiac tissue is tessellated, and the phase index is computed for each mesh face at each time step. By naively assuming the phase map to be continuous, Eq 1 can be calculated by summing phase differences along the edges of each mesh face:


$$\begin{array}{c} I_{C}=\sum_{i\in C}\Delta \phi_{i}, \end{array}$$ (2)

where $\Delta \phi_{i}$ represents the circular difference between $\phi_{i+1}$ and $\phi_{i}$, i.e., the shortest path between two points on the unit circle. For most edges, this circular difference is simply $\Delta \phi_{i}=\phi_{i+1}-\phi_{i}$. However, when the phase jumps from π to −π or vice versa, $\Delta \phi_{i}$ must be adjusted by ±2π to account for the periodicity of the phase. For a closed curve *C*, each phase value $\phi_{i}$ appears twice in the sum: once with a positive sign and once with a negative sign. As a result, the contributions of most edges cancel out, except for those where a phase jump occurs. Assuming the phase map is continuous, only edges with phase jumps will have an absolute phase difference larger than π. A positive jump is recorded when $\phi_{i+1}-\phi_{i}<-\pi$, and a negative jump when $\phi_{i+1}-\phi_{i}>\pi$. The phase index is then given by the difference in positive and negative jumps:


$$\begin{array}{c} I_{C}=n_{+}(C)-n_{-}(C), \end{array}$$ (3)

where *n*_+_(*C*) and *n*_–_(*C*) are the number of positive and negative jumps along *C*, respectively.

Additionally, if a closed curve *C* passes through points with undefined phase, the phase index $I_{C}$ cannot be meaningfully evaluated and is considered undefined.

In this implementation, phase singularities are the smallest closed contours with a non-zero phase index, representing loops that encircle points where the cardiac activity rotates.

#### Phase defects.

Phase defects can be broadly categorized into three types: functional conduction block, structural conduction block, and boundaries, see Fig 2.

**Fig 2:**
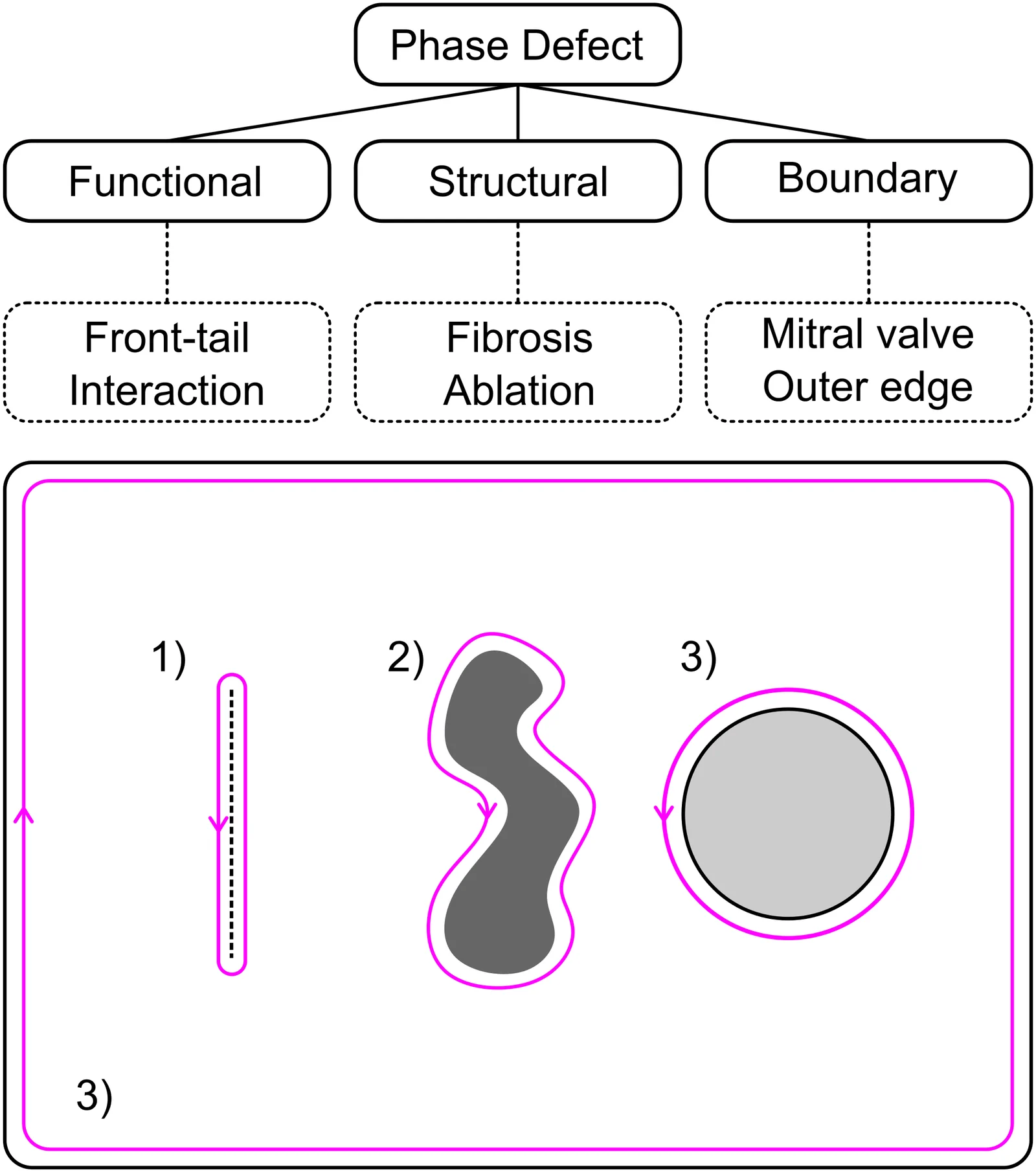
Illustration of functional, structural, and boundary phase defects. Illustration of the three types of phase defects on cardiac tissue: (1) Functional, (2) Structural, and (3) Boundary. A magenta closed curve is shown around each phase defect, indicating the ideal shape of the closed curve C used to calculate the phase index. The orange arrow indicates the direction in which the phase index needs to be integrated, following the approach of [30]. Note that there are two boundaries present: a circular hole, and the outer edge of the rectangular tissue.

First, **functional conduction block** occurs when an excitation wavefront encounters tissue that is still in refractoriness. At such sites, one side of the interface is excited while the other is repolarizing, forming a line of discontinuity in the phase map [18,19].

Second, **structural conduction block** can be caused by the non-conductive structures in the cardiac tissue, such as diffuse fibrosis or dense scar tissue that is created during ablation therapy. In transmembrane voltage maps obtained from simulations, structural conduction block manifests itself as undefined regions in the phase, when the cell voltage does not exceed the activation threshold. However, in electrogram data of optical mapping or electroanatomic mapping, the transmembrane voltage of small regions of cardiac cells gets averaged and smoothed. Therefore, when healthy tissue is surrounded by fibrotic tissue, the resulting electrogram will be affected. As such, structural conduction block would manifest itself as regions of discontinuous phase in electrogram data, akin to functional conduction block.

Third, **boundaries** refer to anatomical or geometric edges in the tissue, such as the borders of a 2D simulation or structures such as the MV, LPV or RPV [31].

To ensure that the phase index is well-defined, curves must avoid crossing discontinuous or undefined regions. Instead, the entire phase defect should be enclosed within the curve, as illustrated in Fig 2. The direction of integration follows the approach of [30], and is indicated in the figure.

#### Extended implementation of phase mapping analysis.

When phase defects are present in the cardiac tissue, the assumption of a continuous phase map is violated, and additional steps are required to ensure that Eq 1 is well-defined.

First, phase defects are identified and excluded from the mesh. To detect discontinuities, the absolute value of the circular phase difference |Δϕ| is evaluated across mesh edges [32]. The rationale is that |Δϕ| remains small where the phase map is continuous, but becomes large when an edge crosses a discontinuity. Faces containing at least one edge where |Δϕ| exceeds a predefined threshold are removed from the mesh, effectively creating holes.

The thresholds for simulated, experimental, and clinical data were hand-selected by visual inspection of the activation maps. The corresponding values were 0.1π, 0.4π, and 0.2π, respectively.

These values were selected to ensure accurate annotation of regions where conduction block was expected.

The “optimal” threshold would be the lowest value for which only the phase defect is removed.

Nevertheless, low threshold values should be used with caution, as they can lead to slow-conducting regions being misclassified as conduction blocks due to the high local phase gradient. In addition, mesh faces containing at least one node with an undefined phase are also removed.

Second, the boundaries of the remaining mesh are extracted to form the closed contours *C* of various sizes, which are then used to evaluate the phase index using Eq 3.

Finally, the remaining mesh is evaluated according to the naive approach that is described above, providing the smallest possible closed contours equal to the mesh faces. In this implementation, we define critical phase defects as the phase defects with a non-zero phase index, which represent regions of rotational activity [20,31].

#### Conservation of total phase index.

In case the phase map on the compact surface *S* contains a finite number of isolated singular points, there exists the following conservation law [30]:


$$\begin{array}{c} \sum_{x_{i}\in S}I(x_{i})+\sum_{H_{j}\in S}I(H_{j})=0 \end{array}$$ (4)

where $x_{i}$ are the singular points in *S*, and $H_{j}$ the holes in the surface *S*. This statement does not hold when discontinuities are present within the phase map [19]. However, it is possible to generalize Eq 4 by treating all these phase defects as holes in a reduced surface $S^{\prime }\subset S$.


$$\begin{array}{c} \sum_{x_{i}\in S^{\prime }}I(x_{i})+\sum_{H_{k}\in S^{\prime }}I(H_{k})=0 \end{array}$$ (5)

where $x_{i}$ are the singular points in $S^{\prime }$, and $H_{k}$ the holes of $S^{\prime }$, including all excised phase defects. From now on we will refer to Eq 5 as the index theorem.

### Study cases

#### Finitewave simulations.

We conducted proof-of-concept simulations on an isotropic 2D grid using Finitewave [33], an open-source Python package for cardiac electrophysiology simulations, based on finite-difference methods, whose transparent implementation of cardiac models facilitates verification of the simulation dynamics.

We used the Fenton-Karma cell model [27] with the MLR-I parameter set, a three-variable phenomenological model suitable for generating phase defects [32] while remaining computationally efficient. Each simulation started from a mesh of 10 ~ cm x 10 ~ cm, with a spatial resolution of 0.25 ~ mm. The tissue was pre-paced with 10 line stimuli (10 ~ cm x 1.25 ~ mm) applied from the left border of the tissue at intervals of 170 ~ ms to establish a stable initial state with an action potential duration of 170 ~ ms. At 1700 ~ ms, the state of the tissue was saved and used as the starting state for the following simulations (see https://doi.org/10.5281/zenodo.17602155).

To investigate distinct activation patterns, we designed three simulation scenarios with specific tissue modifications and additional stimuli, depicted in Fig 3.

- **Linear rotor core.** A single additional square stimulus was applied in the top-left corner of the tissue at 0 ~ ms, so that this stimulus collided with the refractory tissue left by the last planar wave, producing a unidirectional conduction block. This scenario was chosen to illustrate that a linear core may appear as a meandering rotor, and the apparent complexity can be resolved once phase defects are properly accounted for.
- **Parallel activation around a functional conduction block.** Multiple line stimuli (10 ~ cm x 1.25 ~ mm) were applied from the left border at times: 0, 160, 315, 470, 625, 780, 935, 1090, and 1245 ms. To generate a functional conduction block in the tissue, a small central rectangular region of 5 ~ cm by 4 ~ mm was stimulated in between two of the line stimuli at 170 ~ ms. The timing is such that the rectangular region is refractory when the planar stimulus reaches this area, resulting in a conduction block. This simulation represents the phenomenon of an excitation wave encountering a conduction block. This scenario contains no rotation. However, it is a common occurrence in realistic data and therefore worth investigating.
- **Complete and near-complete rotation around scar tissue.** Two circular regions of non-excitable tissue (“scar tissue”) are added to the tissue, one with a radius of 1.2 ~ cm and one with a radius of 1.6 ~ cm and their centers separated by 5 ~ cm. An additional 5 ~ cm x 5 ~ cm stimulus was applied at 0 ~ ms on the left side of the tissue, positioned such that it extends horizontally from the left boundary toward the center of the tissue. The stimulus is vertically centered so that it bridges the gap between both unexcitable regions. The timing of this square stimulus was chosen such that the square stimulus collides with the refractory tissue caused by the last wave, resulting in a unidirectional conduction block, located in the gap between the non-excitable regions. The other side of the square stimulus is excitable, thereby initiating a reentry pair. This scenario illustrates a regular arrhythmia characterized by a complete reentry loop around the smaller, lower obstacle and a near-complete reentry loop around the larger, upper obstacle, representing the dynamics of an atrial tachycardia (AT).

**Fig 3:**
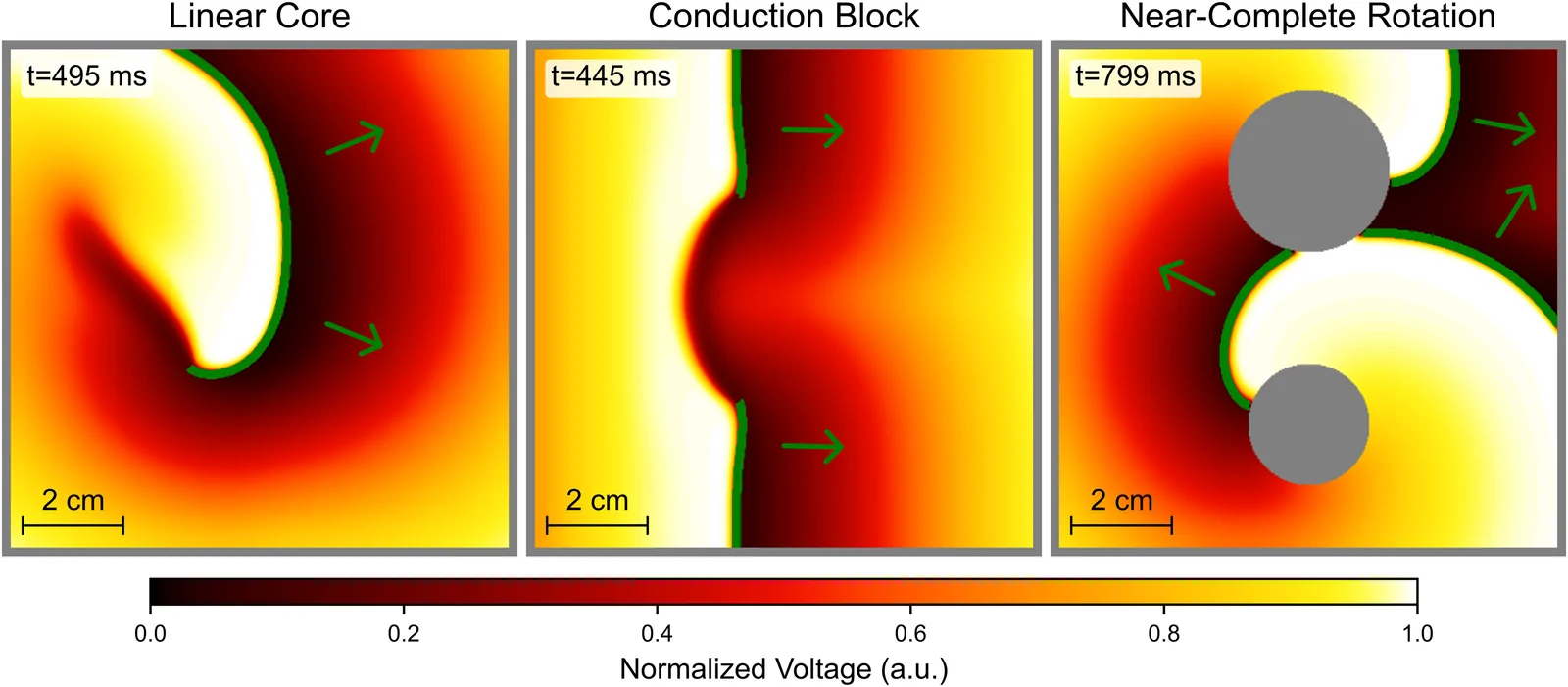
Normalized transmembrane voltage snapshots. Snapshot of the normalized transmembrane voltage in the three simulations (Linear Core, Functional Conduction Block, Near-Complete Rotation). The gray areas indicate unexcitable tissue. The wavefront and its direction is displayed in green.

#### Optical mapping of experimental ventricular fibrillation.

This paper used optical mapping data [34] of ventricular fibrillation in a rat heart that was previously analyzed in [16] (See Fig 4). The data was obtained from [35], where details of heart preparation and ethics are described. We analyzed one case with maximally increased gap junction coupling (80 nM rotigaptide) to test our methods on stable rotors.

**Fig 4:**
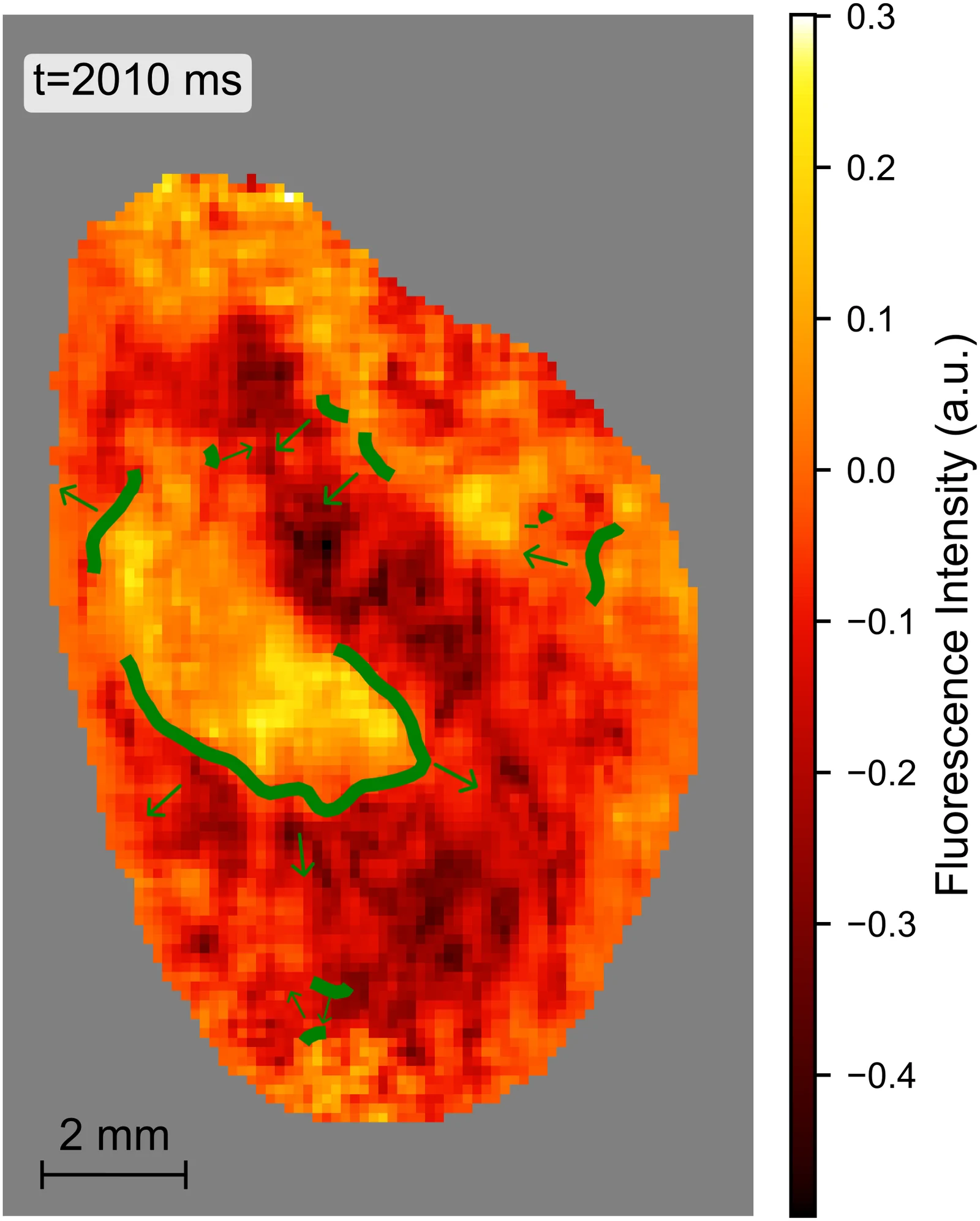
Ventricular fibrillation optical map. Snapshot of an optical intensity map of ventricular fibrillation of the left ventricle of a rat heart acquired in Imperial College London, Great Britain. The gray area indicates the background. The wavefront and its direction is displayed in green.

Briefly, a 250–300 g (9–12 weeks) Sprague–Dawley rat heart was explanted and perfused *ex vivo* via Langendorff perfusion with a Krebs–Henseleit Buffer at $37\pm 0.5^{\circ }\text{C}$ and pH 7.35±0.05. After 15 min, RH237 (40 ~ μL, 5 mg/mL in DMSO) and Blebbistatin (30 ~ μM then 10 ~ μM) were added, plus 80 nM rotigaptide to increase gap junction coupling. VF was induced electrically at the left ventricle base (MicroPace, USA) and maintained with Pinacidil (30 μM). Optical mapping used RH237 excited at 530 nm; emission was recorded by high-resolution CMOS cameras at 1 ~ kHz, 128×80 pixels, and 100–125 μm pixel width.

Data were preprocessed in MATLAB, using: background removal, 3×3 binning, 50-order FIR low-pass at 100 Hz, and 100 ~ *ms* window drift removal. Further details on the signal processing protocol can be found in [28]. Afterward, the phases were smoothed by transformation to the complex vectors $\vec{z}$ (Eq 6), and smoothing those complex vectors using a Gaussian kernel with a standard deviation of 1 pixel (100–125 μm). Finally, the resulting vectors were converted back to phases.


$$\begin{array}{c} \vec{z}=e^{i\phi } \end{array}$$ (6)

It is important to note that the measured optical intensity averages and smooths the transmembrane voltages of small regions of cardiac cells, similar to the electrogram.

#### CARTO map of clinical atrial tachycardia.

The final case study is a unipolar electroanatomic map from a clinical left-atrial atrial tachycardia (AT) case recorded with the CARTO (Biosense Webster, Johnson & Johnson) system at AZ Sint-Jan Hospital, Bruges (octarray catheter), and shown in Fig 5. The map included three major anatomical boundaries: the mitral valve (MV), and the left and right pulmonary veins (LPV and RPV). This retrospective study had patient consent for scientific use. The case had the following properties:

- Whole-atrium coverage with local point density > 0.4 points/cm^2^
- Mean electrode–mesh distance < 4 mm
- Known locations of all anatomical boundaries
- Previous unsuccessful ablation of the mitral isthmus (between the MV and LPV) and the vein of Marshall
- Confirmed diagnosis of reentry around the MV + LPV and anterior scar
- Successful ablation between an anterior scar and MV, terminating the tachycardia
- Known tachycardia cycle length (TCL) before and after ablation: 317 ms to 0 ms

**Fig 5:**
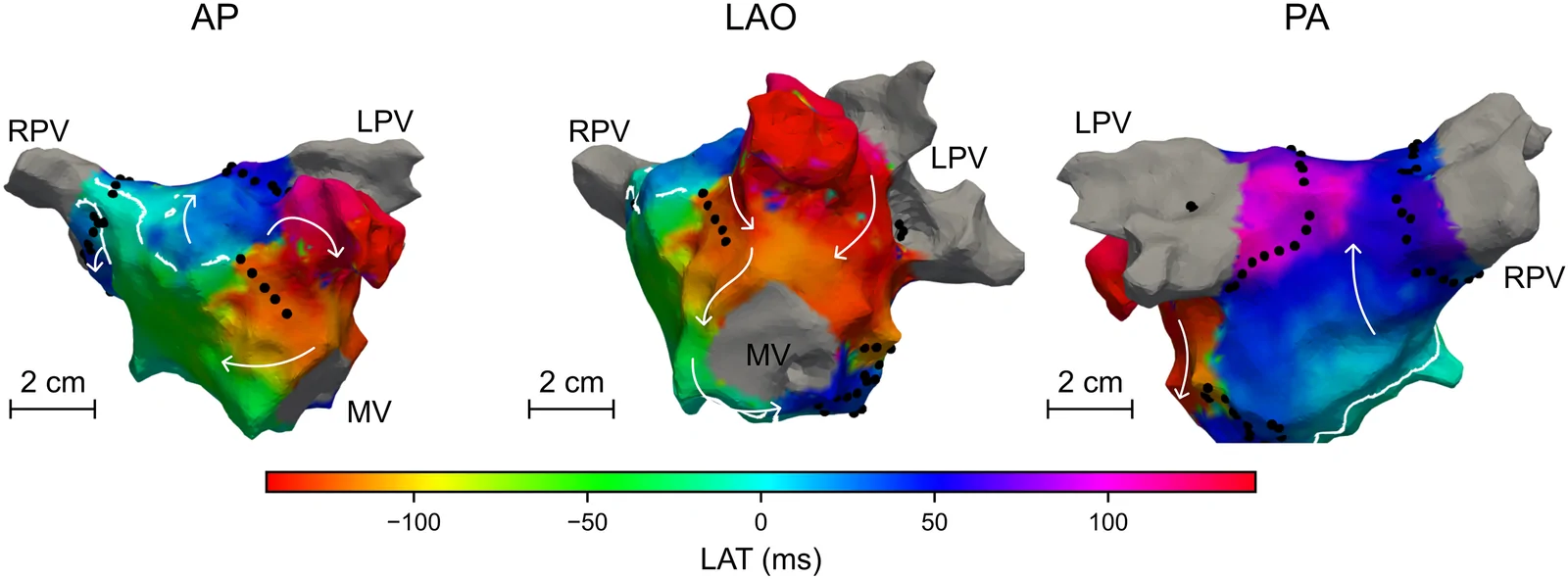
Left atrial tachycardia activation map. CARTO activation map of a clinical atrial tachycardia case of the left atrium of a patient in AZ Sint-Jan Bruges, Belgium. The depicted viewpoints are from left to right: Antero-Posterior (AP), Left Anterior Oblique (LAO), and Postero-Anterior (PA). The gray areas indicate the anatomical valves. The wavefront and its direction are displayed in white.

The CARTO activation map data format consists of a triangular mesh of the atrium surface, consisting of points and triangles. Additionally, the scalar values on each point correspond to the interpolated local activation time (LAT) value based on the measured LAT values from the nearby electrodes. Due to the highly varying LAT values near the wavefront line, interpolation artifacts arose in the electroanatomical map. Therefore, the LAT map was preprocessed before analysis via conversion to complex vectors *z* (Eq 6) as described in the previous section, where the kernel standard deviation was 1 ~ *mm*, and the phases are calculated via:


$$\begin{array}{c} \phi =2\pi \frac{\text{LAT}}{\text{TCL}} \end{array}$$ (7)

## Results

### Finitewave simulations

To illustrate the key features of each simulation, results are shown for a representative single time frame. The full analysis code [36] and https://doi.org/10.5281/zenodo.17602155 are provided, enabling inspection of other time frames.

**Linear rotor core.** Fig 6 shows a simulation of functional reentry, with a core that displays distinct rotational activity around a phase defect. The absence of non-conducting regions in the action potential field suggests that the defect represents a functional conduction block. The naive approach identifies on average two positive and three negative phase singularities at the central defect per time frame (Fig 9). In contrast, the extended approach finds two critical phase defects per time frame: the central defect a phase index of minus one, and the outer boundary with a phase index of one (Fig 9). Moreover, the extended approach adheres to the index theorem for all time frames in contrast to the naive approach (Fig 10) This result aligns with the underlying tissue dynamics: namely, a single central rotation, which is also clearly visible in https://doi.org/10.5281/zenodo.17602155.

**Fig 6:**
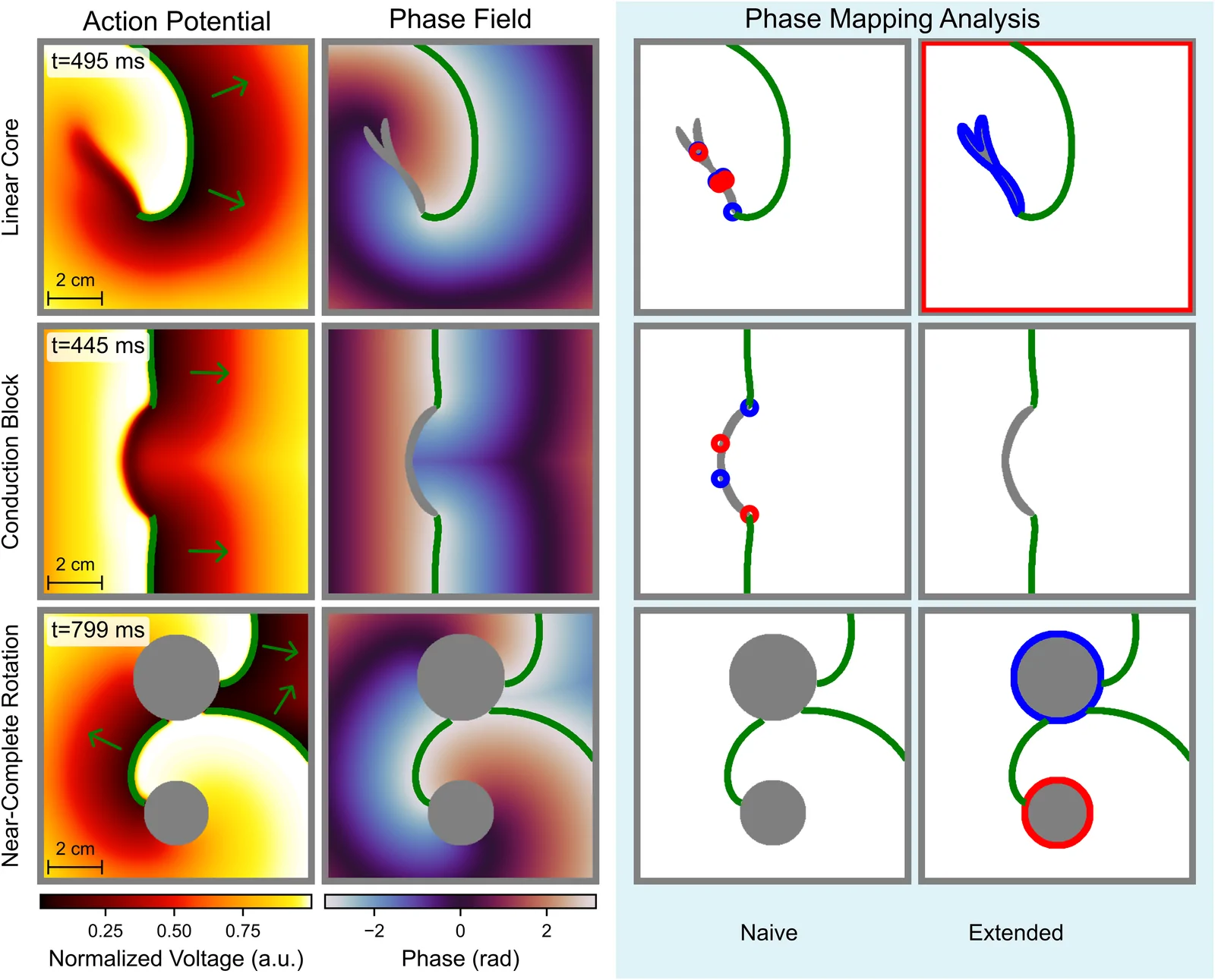
Phase mapping analysis of simulations. Snapshots of simulations of various patterns using the Fenton-Karma model. The patterns are from top to bottom: A linear rotor, a functional conduction block without rotation and a reentry-pair with near-complete rotation. The wave front and its direction is displayed in green, and the phase defects are annotated in gray. *First column*: Normalized action potential. *Second column*: The phase map constructed from the action potential. *Third column*: The results of the naive phase mapping. The blue and red circles denote phase singularities with phase indices of minus and one, respectively. *Fourth column*: The results of extended phase mapping. The blue and red lines denote closed curves with phase indices of minus one (clockwise rotation) and one (counter-clockwise rotation), respectively.

**Parallel activation around a functional conduction block.** Fig 6 illustrates a functional conduction block in the absence of rotational activity. As in the previous case, the action potential field shows no non-conducting regions, indicating that the phase defect is again a functional conduction block. Despite the lack of rotation, the naive approach detects an average of six positive and six negative phase singularities around the defect per time frame (Fig 9). In contrast, the extended approach finds only a non-critical phase defect at the center for all time frames (Fig 9). Additionally, the phase index is conserved for all time frames in the extended approach, in contrast to the naive approach (Fig 10). Given that the simulation exhibits no rotations, as evident from both the snapshot and https://doi.org/10.5281/zenodo.17602155, extended phase mapping is the only approach that produces results consistent with the non-rotational nature of the simulation.

**Complete and near-complete rotation around scar tissue.** Finally, Fig 6 shows two simultaneous rotations around obstacles of different sizes. Unlike the previous simulation, the phase defects in this case are structural conduction blocks, which are visible in both the action potential field and the phase map. The snapshot and https://doi.org/10.5281/zenodo.17602155 demonstrate that the smaller obstacle supports a complete rotation, while the larger scar shows a near-complete rotation that is interrupted before the wavefront can fully circle the obstacle. The naive approach generally detects zero phase singularities per time frame, since the phase within scar tissue is undefined (Fig 9).

Occasionally, tissue adjacent to the scar briefly remains refractory, forming a small functional block attached to the structural one. The naive approach can then detect a phase singularity at this location.

The extended approach identifies two critical phase defects per time frame: the smaller one receives a phase index of one, and the larger one a phase index of minus one (Fig 9). Additionally, the tissue boundary is identified as a non-critical phase defect. Furthermore, the extended approach adheres to the index theorem for all time frames in contrast to the naive approach.

### Optical mapping of ventricular fibrillation

The differences between the naive and extended phase mapping approaches become even more pronounced when applied to optical mapping data of ventricular fibrillation, illustrated in Fig 7 and https://doi.org/10.5281/zenodo.17602155. This case is characterized by multiple activation wavefronts interacting with each other, hosting both stable and unstable rotational centers, characteristic of fibrillation. The naive approach produced an average of 19 positive and negative static and meandering phase singularities per time frame, obscuring the underlying activation pattern. In contrast, the extended approach clarified the analysis by detecting an average of 6 positive and negative critical phase defects per time frame (Fig 9). Furthermore, the naive approach adhered the index theorem for 18.47% of the recorded frames in contrast to 100% for the extended approach (Fig 10). Additionally, the extended approach discovered the presence of critical phase defects hosting more than one wavefront. Moreover, the phase defect containing the outer edge occasionally had an absolute phase index larger than one due to the presence of multiple wavefronts.

**Fig 7:**
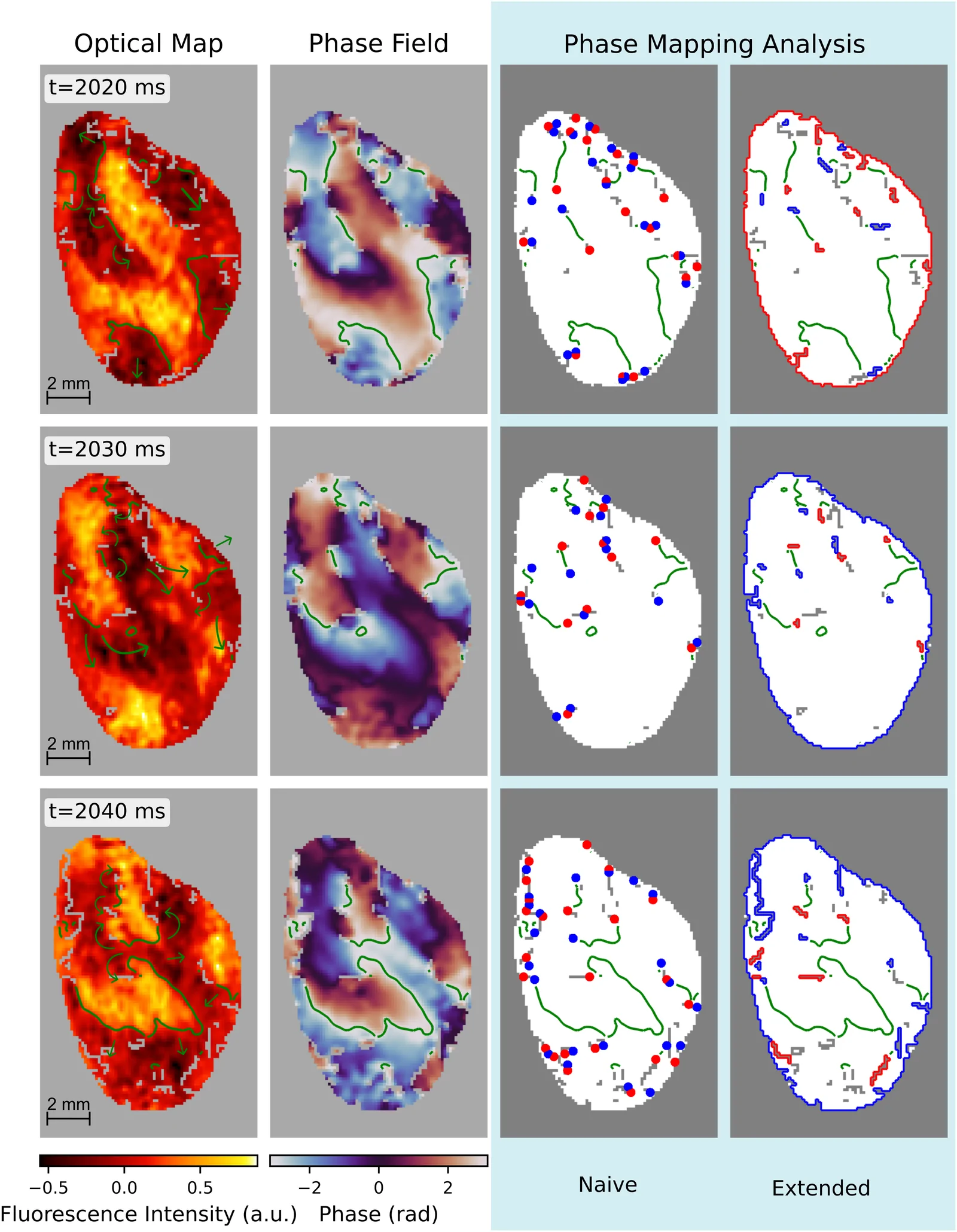
Phase mapping analysis of an optical mapping. Several snapshots of an optical intensity map of ventricular fibrillation of the left ventricle of a rat heart acquired in Imperial College London, Great Britain. The wave front is annotated in green and the phase defects are annotated in gray. *First column*: Optical mapping displaying the fluorescence intensity. *Second column*: The phase map constructed from the LAT map. *Third column*: The results of the naive phase mapping. The blue and red spheres denote phase singularities with phase indices of minus and one, respectively. *Fourth column*: The results of extended phase mapping. The blue and red lines denote closed curves with phase indices of minus one (clockwise rotation) and one (counter-clockwise rotation), respectively.

### CARTO map of clinical atrial tachycardia

The clinical case in Fig 8, https://doi.org/10.5281/zenodo.17602155 clearly illustrates several key differences between the naive and extended phase mapping approaches. This case was clinically diagnosed as atrial tachycardia sustained by reentry around the MV + LPV and a nearby scar line. The tachycardia was terminated via ablation between the MV and the scar. The naive approach detects 11 positive and 12 negative static phase singularities in the proximity of the phase defects as detected by the extended method

**Fig 8:**
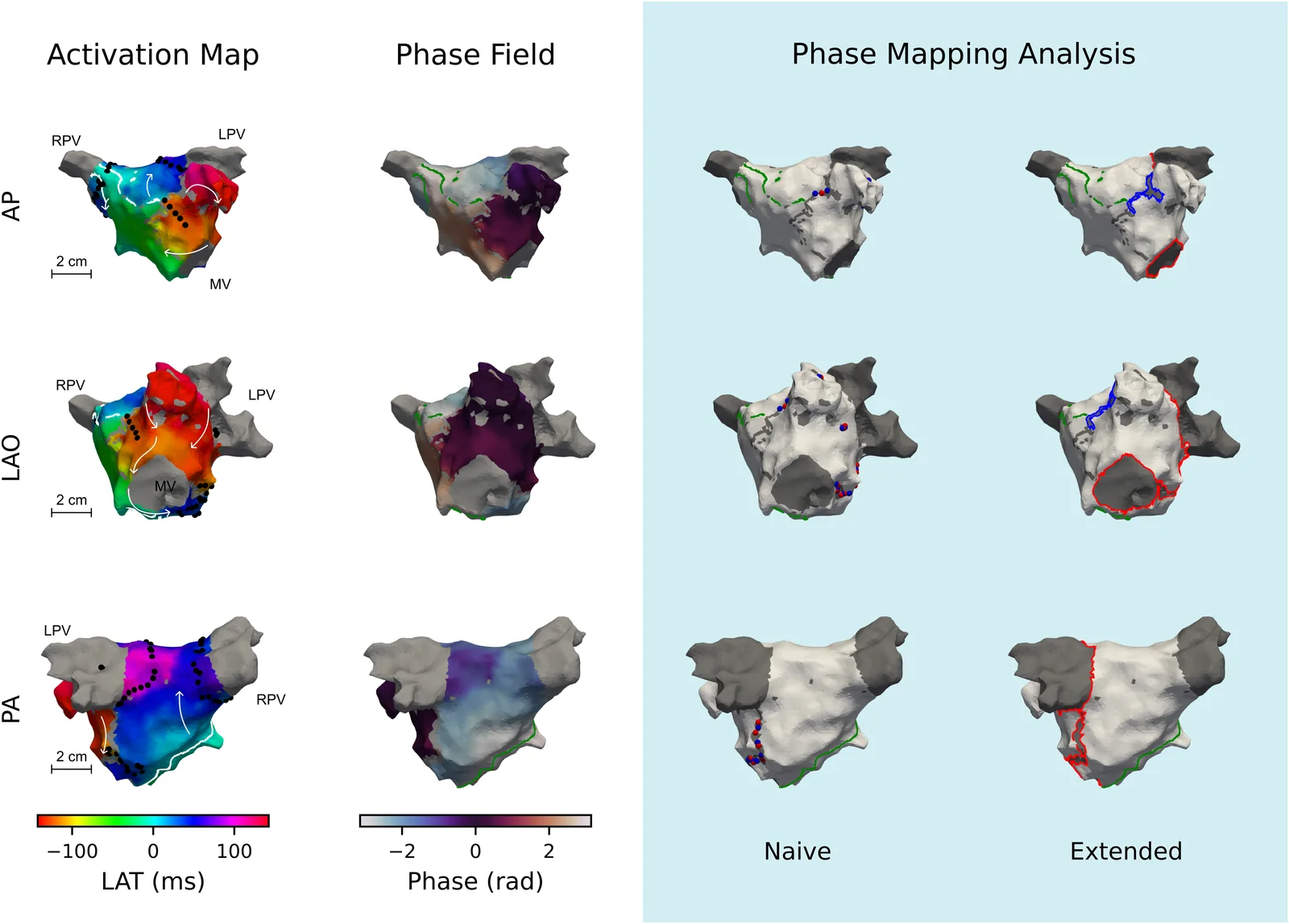
Phase mapping analysis of a clinical atrial tachycardia. A clinical CARTO map of atrial tachycardia diagnosed with reentry around the MV + LPV and a nearby scar. The wave front and its direction is displayed in white on the activation map and green on the other columns, the phase defects are annotated in gray, and the ablation points are annotated in black. *First panel*: Activation map. *Second panel*: The phase map constructed from the LAT map. *Third panel*: The results of the naive phase mapping. The blue and red spheres denote phase singularities with phase indices of minus and one, respectively. *Fourth panel*: The results of extended phase mapping. The blue and red lines denote closed curves with phase indices of minus one (clockwise rotation) and one (counter-clockwise rotation), respectively.

(Fig 9). The anatomical valves, including the MV and LPV, are also completely devoid of any detections,

**Fig 9:**
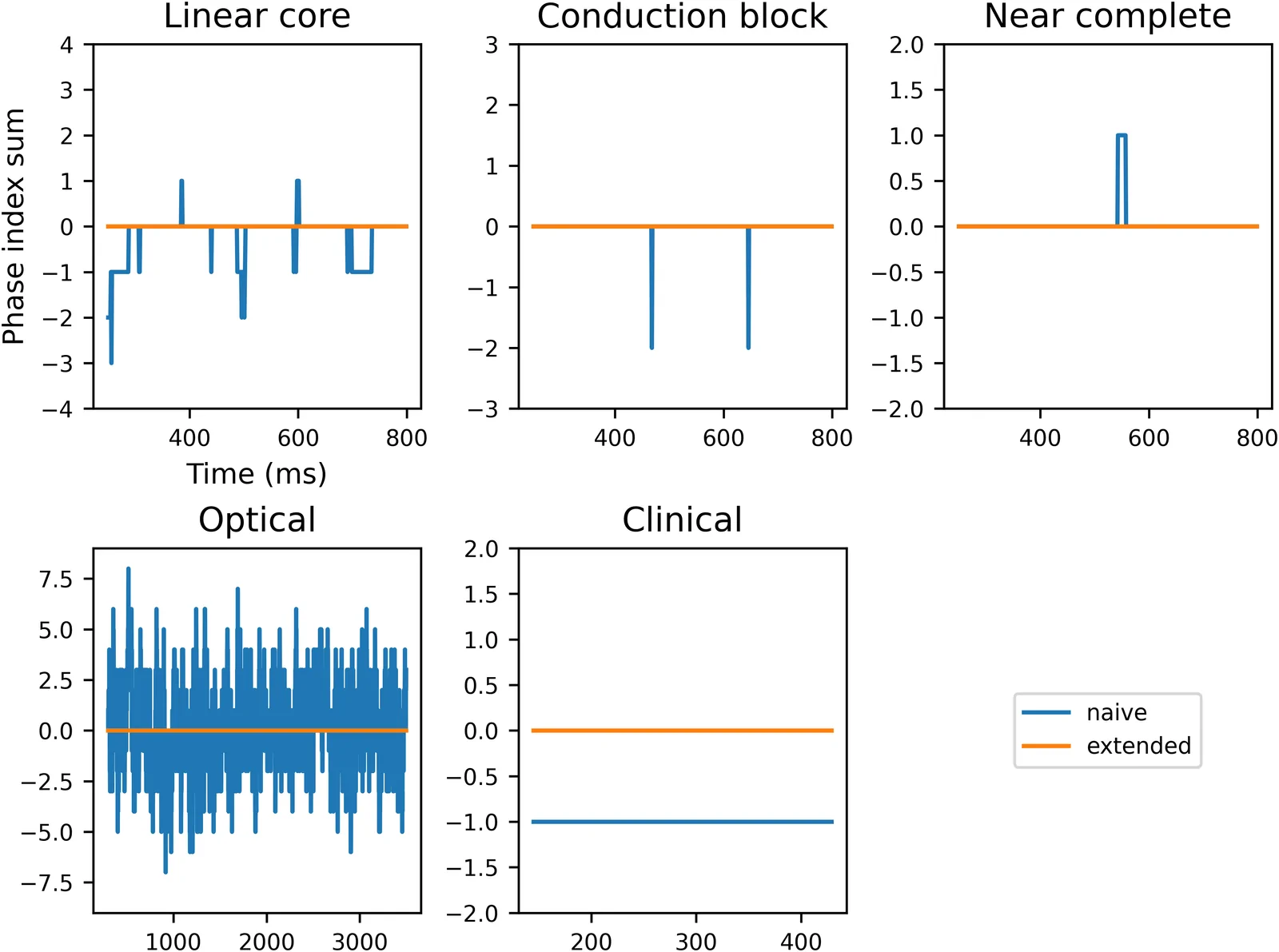
Number of detected phase singularities (naive method), and critical phase defects (extended method) over time for all five datasets. The naive approach has a higher number of detections and greater variability over time compared to the extended approach.

In contrast, the extended approach shows a different result. One positive and negative static critical phase defect were consistently detected around the MV + LPV and a nearby phase defect line respectively (Fig 9). Additionally, 35 static non-critical phase defects are consistently annotated, indicating the presence of conduction block. In particular, the previous ablation line between the MV and LPV was correctly annotated as a conduction block. These results are in line with the diagnosis, where the MV + LPV and an anterior scar were found to host reentry. Additionally, this agrees with the index theorem previously described in [20,31], stating that the sum of all phase indices must equal zero. This is in contrast to the naive approach, which violates the index theorem for every time frame of the recording (Fig 10).

**Fig 10:**
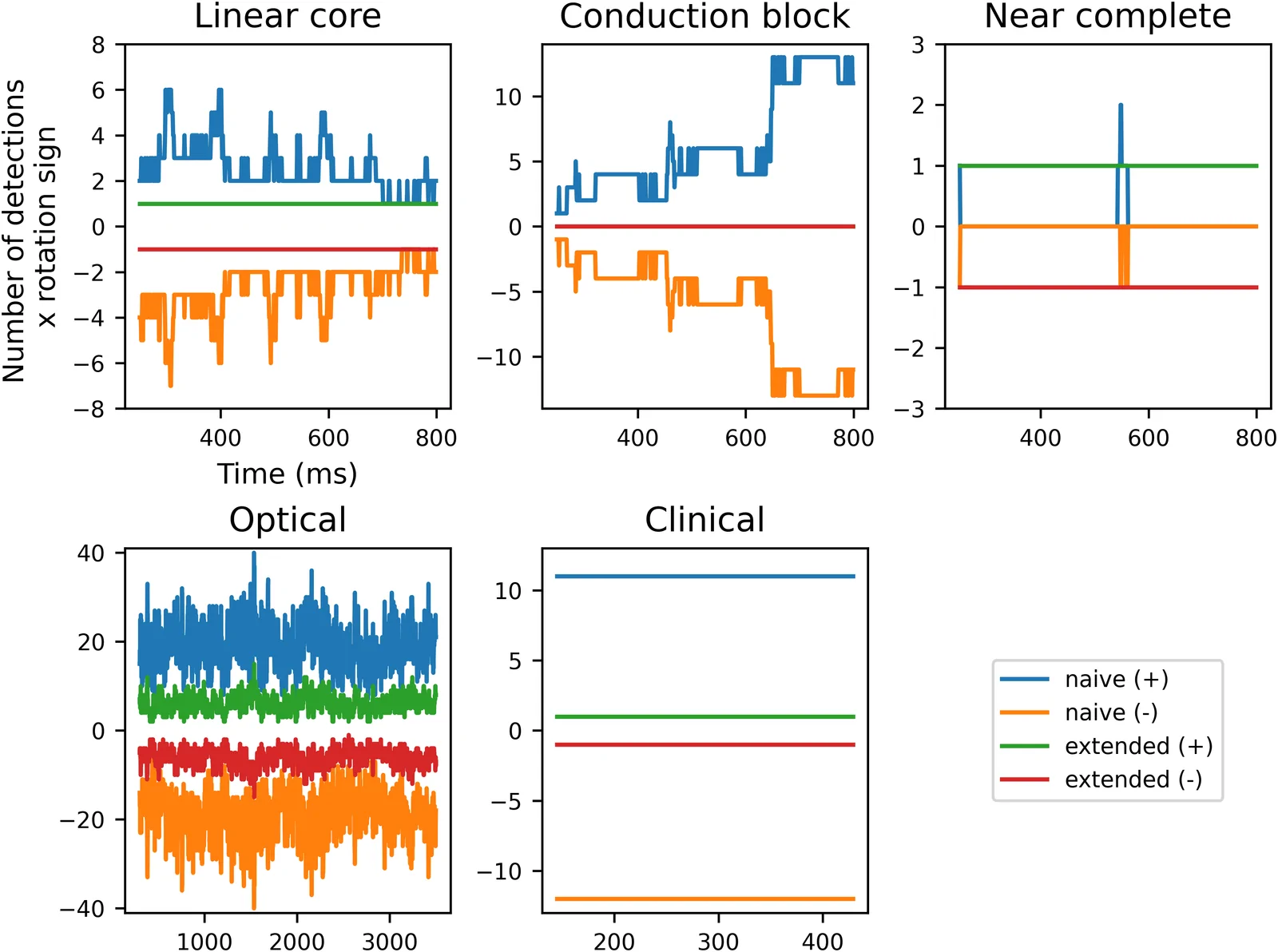
Phase index sum of phase singularities (naive method), and critical phase defects (extended method) over time for all five datasets. The naive approach violates the index theorem, while the extended approach adheres to the index theorem for all time frames.

## Discussion

### Main findings

The three simulations in Fig 6 illustrates a consistent failure of the naive approach: false positive phase singularities are detected whenever a functional conduction block is present. These false detections could arguably be filtered out by clustering [15,37,38] or by requiring a minimum duration for a singular point to persist [37]. However, the naive approach also fails to detect near-complete rotations around large obstacles due to the undefined phase of scar tissue, and a minimum-duration filter would still discard such near-complete rotations. Consequently, when an activation map contains multiple types of phase defects, no straightforward post-processing strategy exists to resolve these competing errors. Most importantly, the root cause is not noise, but rather that the phase index is fundamentally ill-defined in the presence of phase defects. The extended approach directly addresses both failures by computing the phase index around phase defects rather than through them, thereby eliminating false positives that arise from functional conduction blocks while simultaneously identifying near-complete rotations, as demonstrated in the third simulation. The latter is of particular clinical relevance: recent studies have shown that near-complete rotation can play a critical role in guiding ablation strategies [20,39,40].

The optical mapping and clinical data reinforce this picture. In both cases, singular points are consistently attached to phase defects, suggesting they reflect the presence of a defect rather than genuine rotational activity. For the optical mapping data, the naive approach implicitly assumes a continuous phase, causing wavefront endpoints attached to functional conduction blocks to appear as spurious rotational drivers. Our method shifts the paradigm from rotation around singular points to rotation around two-dimensional phase defects, inherently eliminating these false positives rather than filtering them in post-processing. For the clinical map, the naive approach fails to detect the reentry around the anterior scar and the near-complete rotation around the MV and LPV. The extended approach, by contrast, demonstrates the full interpretive value of phase mapping: false positives are filtered out, complete rotation is detected around the anterior scar, and near-complete rotation is identified around the MV and LPV.

Taken together, these results highlight a fundamental limitation of the naive approach: it neither reliably detects rotational activity nor adheres to the index theorem, which requires the sum of phase singularity indices over a compact surface to equal zero. Violations of this constraint are observed across all datasets, reflecting the ill-defined nature of the phase index in the presence of phase defects. The extended approach, by computing the phase index around rather than through phase defects, restores theoretical consistency: the index theorem is satisfied in all cases, and the detected singularities carry genuine interpretive value as markers of rotational activity.

Beyond theoretical consistency, the extended approach offers a conceptually distinct alternative to conventional false-positive suppression. Existing post-processing strategies such as phase singularity clustering or minimum-duration filters implicitly assume phase continuity and rely on heuristic parameters that lack direct physiological interpretation. The extended approach instead grounds false-positive suppression in the mathematics of the phase index: by explicitly modeling phase discontinuities, every detection corresponds to a complete or near-complete rotation around a phase defect, eliminating the need for arbitrary post-processing. Additionally, the extended approach also avoids the false negatives that the naive approach introduces near regions of undefined phase, such as scar tissue or anatomical boundaries. While the phase-difference threshold used here provides a practical means of identifying discontinuities, more sophisticated characterizations of phase continuity remain an avenue for future work.

### Previous research

This paper is closely tied to previous research concerning the detection of phase defects and rotational drivers.

Several previous studies have addressed the role of phase defects in the analysis of activation maps. Notably, the authors of Tomii et al., Arno et al., and Kabus et al. [18,19,32] emphasized the importance of phase defects when interpreting phase maps.

In particular, in Kabus et al. [32], the authors pointed out that the phase index is ill-defined in the proximity of phase defects, implying a limitation of naive phase mapping. The results in Fig 6 indicate the importance of assigning a phase index to phase defects to distinguish between parallel activation and rotational activity.

Furthermore, in Tommi et al. [18], the authors argue that the center of a spiral wave is more appropriately interpreted as a phase defect with a finite circumference rather than as a singular point. Likewise, our results support this interpretation: once the interactions between the wavefront and the refractory tail are properly accounted for by detecting phase defects, the apparent singular points vanish, as illustrated in Fig 6.

The influence of phase defects has been indirectly addressed in previous studies, such as Li et al. [15] and Wieser et al. [38], which identified phase jumps by setting the phase-difference filter threshold above π. This approach effectively filters out the fraction of conduction blocks with an absolute phase difference above π, but it may still handle large non-conducting regions and boundaries improperly. Moreover, detecting phase defects and phase jumps in a single step, restricts the use of additional features to improve phase defect detection.

In related work, several methods have been proposed to characterize the nature of non-excitable structures [39, 40]. Here, the authors reported observations of critical anatomical features exhibiting patterns similar to those shown in Fig 6. However, more notably, in Van Den Abeele et al. and Duytschaever et al. [20,31] employed the same phase index used in this manuscript to assess the criticality of anatomical boundaries (e.g., MV, LPV, RPV). Building on this research, we extend the approach by incorporating all kinds of phase defects.

Beyond the scope of this work, previous studies have characterized meandering patterns of spiral waves, often described as epitrochoid or hypotrochoid trajectories [41,42]. While this work centers around phase defects and leans into the hypothesis that singular points are not representative for rotational activity, it does not necessarily invalidate such studies. However, it would be more suitable to use wavefront endpoints instead of singular points for such studies. Assuming these endpoints are always attached to a phase defect [18,19], studying how phase defects evolve over time offers a new way for characterizing such patterns. For example, the simulation of a linear rotor core (see https://doi.org/10.5281/zenodo.17602155) shows an elongated defect around which the wavefront rotates. The phase defect itself also rotates, but at a slower rate than the wavefront rotating around it. In this case the meandering pattern can be described by the size and rotational speed of the phase defect, and the rotational speed of the wavefront endpoint around the defect. These variables should in principle be sufficient to describe epitrochoid or hypotrochoid trajectories [42].

### Limitations and future prospects

The primary aim of this work is to highlight the challenges that arise when phase defects are present and to demonstrate the potential benefits of explicitly addressing them during phase mapping analysis. As such, this study should not be viewed as a detailed statistical analysis on a large dataset investigating phase defect detection algorithms and robustness. We will now describe four limitations that are tied to this.

First, the method used to detect phase defects in this study, relying on one parameter, is relatively basic and intended primarily for demonstration purposes.

Because the thresholds were hand-selected, there is no guarantee that the chosen values are optimal.

To make phase mapping more reliable and robust for interpreting realistic data, more sophisticated approaches relying on more features of the electrogram for phase defect detection should be explored. For example, features such as low voltage, mid-diastolic potentials, after-depolarizations, double potentials, fractionation, and other electrogram characteristics associated with decreased cardiac conduction could provide more robust indicators of phase defects and are valuable future research topics [43,44].

Furthermore, methods that leverage machine learning could also be useful to identify phase defects as an input to phase mapping, without replacing the phase mapping analysis itself [45,46].

Second, the size of our dataset is severely limited to five cases. The simulations used in this work are also intentionally simplified and do not reflect realistic cardiac tissue. The reason for this was to isolate the core problems with the naive implementation of phase mapping, removing additional effects such as high signal noise and low mesh resolution. This limitation is partly mitigated by including the analysis of the optical map and clinical activation map, reflecting more realistic scenarios. Our research group is currently working on analyzing larger datasets of atrial and ventricular tachycardia using extended phase mapping to obtain more statistically significant and robust results.

Third, the effects of signal noise and mesh resolution were not investigated in this study. The effect of these factors on phase mapping has been demonstrated in previous studies [7,8,14]. In this work, all simulations contained a very high resolution and no signal noise; therefore, we excluded the effects of these factors on the interpretations of these results. This could not be done for the experimental data, as the optical map contained a relatively low resolution and considerable signal noise [35], possibly impacting our results. Similarly, the clinical activation map had a high resolution, but contained interpolation artifacts that manifested themselves as noise in the resulting phase map. However, we have included some preliminary results in the appendix (S1 and S2 Figs), illustrating that Gaussian noise on the phase map manifests itself as phase defects for our implementation relying on phase differences. For noise levels of above σ=0.2π, the naive method (threshold = π) breaks down as multiple false positive phase singularities are found. The extended approach instead converts false positives into phase defects by lowering the phase difference threshold. Consequently, we acknowledge the effect of signal noise and resolution on our results, and propose their investigation as a valuable future research topic.

Fourth, the influence of the definition of phase on our results was not investigated. In this paper, we refrained from using the conventional phase definition based on the Hilbert Transform due to erroneous phase defect detections near wavefronts [32], leading to incorrect interpretations. As such, we opted for a modified definition of phase recommended by [32] based on an activation threshold and consecutive activation times, yielding a sawtooth-shaped phase. We acknowledge the influence of this activation threshold on the interpretation of our results, and leave the investigation of its influence for future studies.

Beyond the choice of activation threshold, both the Hilbert Transform and sawtooth phase definitions break down near phase defects. This can be seen in Fig 6 where the defect in the linear core simulation has branched structure. The branches at the rotor core are an artifact of phase mapping, where linear interpolation between sequential LATs breaks down near phase defects due to aperiodicity. Since the artifact mimics the signature of a true phase defect, it can lead to false-positive detections: phase singularities in the naive approach and incorrectly shaped phase defects in the extended approach. It should therefore be interpreted with caution.

Finally, the methods in this paper are only applicable for two-dimensional surfaces with boundaries, which are topologically equivalent to n-holed spheres. We do not handle more complex geometries present in bi-atrial re-entry or ventricular tachycardia for example. However, we are currently working on a novel topological framework that generalizes this work for arbitrary topological surfaces. In this work, boundaries are redefined as the two-dimensional closed surfaces that bound phase defect volumes, contrary to the one-dimensional loops that bound phase defect surfaces in this work. Preliminary results show that from these boundaries, the extraction of critical re-entry circuits in bi-atrial re-entry and ventricular tachycardia becomes possible.

## Conclusion

We demonstrated the influence of phase defects on the detection of phase singularities using a naive phase mapping implementation across five case studies encompassing simulated, experimental, and clinical data.

We found that the naive method produces both false positive and false negative detections, making the interpretation of wave patterns inconsistent and more convoluted.

In contrast, the extended approach provides consistent results and interprets the drivers of rotation as phase defects with a non-zero phase index.

These findings highlight the limitations of using singular points to characterize wave patterns, particularly in clinical settings where near-complete rotations around large obstacles are critical targets for ablation therapy.

Instead, we propose that characterizing arrhythmia drivers as complete or near-complete rotation around phase defects provides a more complete picture than singular points alone.

Rather than promoting our specific implementation, we emphasize the necessity of explicitly addressing phase defects in phase mapping analyzes. We hope this work motivates further research into the role of phase defects and encourages their integration into analytical workflows.

## Supporting information

S1 FigIllustration of naive and extended phase mapping for an S1 simulation on a 400x400 grid with increasing levels of Gaussian noise.The naive method breaks down for noise with a standard deviation of $\sigma_{\text{noise}}=0.25\pi$ as multiple false positive detections are found. The extended approach (with a phase difference threshold of 0.5π) is robust against all levels of noise as no critical phase defects are found.(TIF)

S2 FigIllustration of the effect of noise and the phase difference threshold on the number of false positive detections in an S1 simulation.Note that a phase difference threshold of π closely resembles the naive approach, with the inclusion of the boundary phase defect. Decreasing the phase difference threshold converts the false positive critical boundaries to false positive phase defects. The naive approach breaks down for noise levels above 0.2π, while the extended approach contains no false positive critical boundaries for all levels of noise for a phase difference threshold below 0.5π.(TIF)
