# Supplementary material for: Going around phase defects: Addressing a fundamental flaw of phase singularity detection
Source: PLoS Comput Biol. 2026 Oct 13;22(10):e1013963. doi: 10.1371/journal.pcbi.1013963 (PMC13705599; doi:10.1371/journal.pcbi.1013963)
Supplement: Supplementary file 1 [file pcbi.1013963.s003.pdf]

I thank the authors for presenting a practical and elegant potential solution to phase defects during phase mapping. There is a clear demonstration of how the extended method can make phase maps more interpretable and reliable in a range of relevant scenarios. My main concern is with the selection of the circular phase difference threshold $|\Delta\phi|$ (I will call it CPDT) used to remove phase defects.

My reading is that, once the phase map is constructed, the method for detecting and removing phase defects effectively hinges on the choice of CPDT. It was briefly mentioned that the CPDT must be carefully selected as to not misidentify defects, such as identifying very slow conduction as block. There was no expansion on exactly how these were chosen for the study nor how one might go about deciding this threshold for different data. They seem to have been hand-selected based on manually reviewing the data, implying an assumption or prior knowledge of areas containing defects, especially in the case of functional block. Please expand this section of Methods to both describe exactly how you chose the thresholds in this study and provide a principled approach to selecting thresholds for new data sets. I appreciate an 'optimal' way of identifying and removing defects would use more than a single threshold parameter, but given that only one parameter is presented, the manuscript is currently lacking in explanation and exploration of it.

Just as a check on my understanding, would a CPDT of 2π effectively reduce the extended method to the naïve case, as the maximal circular difference would be permitted thus no defects are removed?

How is the outer boundary defined on a complex 3D geometry – is it the largest geodesic that traverses the entirety of the mesh? Relatedly, all the examples presented are of a "single boundary" domain; how would the outer boundary be defined for multiple geometries that "go together" like you might see in a bi-atrial circuit (where the LA and RA meshes would both need consideration)? Could the phase map be continuous through the septum, or would you need to interpret 2 separate phase maps? Clinically mapped meshes like in CARTO are usually multiple separate surface meshes registered in the same space, so how is the outer boundary defined – separately for each mesh, or together?

Even after flicking through the manuscript referenced for it being "convention", it is not immediately obvious to me why the outer edge is integrated in the opposite direction (e.g in Fig 5) – from reading your Methods it seems to be to distinguish it from the inner edges – is that correct?

Outer edge absolute phase index > 1 due to multiple wavefronts is interesting; does it also imply the wavefronts are travelling in opposing directions relative to the direction of integration of the outer edge?

How is conduction block due to wavefront collisions treated – would the defects effectively trace a path of defects caused by the wavefront collisions, visualising a kind of “isthmus” where the boundaries creating the isthmus are mesh holes that have been removed?

The CARTO meshes I work with are usually ~1mm resolution, but yours is ~0.4mm – was this refined and re-interpolated, or was it taken directly from CARTO?

How long do the extended phase maps take to compute? Is it feasible to compute the phase map, or to recompute with different parameters e.g updating CPDT, in near-real time? Must they be computed after an entire map has been collected e.g a full chamber LAT map?

I think re-organising the current Methods subsections to have the following structure makes for a more logical reading order: phase mapping fundamentals (Phase mapping); phase defects and the extended phase mapping approach to remedy them; simulation/experimental/clinical setups used for demonstration (Study cases).

The results section is wanting for a graphical representation of the comparison between naïve and extended phase mapping. A list of categories to show include but are not limited to: #phase defects (removed/remaining in the extended case, if there are phase defects remaining above the threshold that were not removed), #true rotors missed, #false rotors detected. A stacked bar plot with the bars colour-coded with “simulation”, “experimental”, “clinical” might work as a good visual representation, although this is just a suggestion, and you may find a different visual representation has more explanatory power.

Figures 6-9 could be combined into one figure – there is a lot of repetition in the captions, and the figure layout and colours are nice and consistent, so it makes sense to combine them into a single figure and put the snapshot time in the row.

Out of interest, what would happen if there was a phase defect within the midmyocardium? The atrial free wall is thin but what about thicker areas like Bachmann’s bundle which may participate in bi-atrial circuits? For a volumetric mesh, could you bound the defects by a surface instead of a curve to identify phase defects and remove volumes? I appreciate this can get complicated and is almost certainly out of scope for the manuscript.
