# Supplementary material for: Going around phase defects: Addressing a fundamental flaw of phase singularity detection
Source: PLoS Comput Biol. 2026 Oct 13;22(10):e1013963. doi: 10.1371/journal.pcbi.1013963 (PMC13705599; doi:10.1371/journal.pcbi.1013963)
Supplement: Supplementary file 2 [file pcbi.1013963.s005.pdf]

EPM paper review

April 2026

We thank all three reviewers for their careful reading of this manuscript and for their valuable comments. We hope that we have addressed all points raised and believe that the paper has been significantly improved thanks to their constructive feedback.

1 REVIEWER 1

1. *The reviewer's only major question is how valid is Eqn 5 when interpreting 3D data on a surface. This does not detract from its usefulness in 2D. In 3D, phase singularities can be I, U, or O-shaped filaments which may or may not have end points on the measured surface. This is particularly pertinent for ventricles, but may also be an issue with left atrial with high endo- and epi dissociation. Does the theory hold that charge is neutral for manifolds sampled from the 3D space?*

   answer: We appreciate the reviewer's valuable question. Although investigating rotational activity in 3D tissue is an active area of our current research, it falls outside the scope of this paper. Nevertheless, we are happy to address your question here to the best of our ability.

   In 2D, the index theorem holds for all compact surfaces (closed surfaces with boundary loops), which states that the sum of topological charges of all boundary loops must equal zero [5]. An example of such a surface is a 3-holed sphere representing the right atrium. In 3D, we have applied the index theorem to compact surfaces sampled from a volume [17]. However, it remained unclear which surfaces should be probed to capture all rotational activity.

   To solve this issue, we redefined boundaries as the closed surfaces of healthy tissue that bound phase defect volumes and claim that all rotational activity is captured on these surfaces. The fundamental theorem in homology states that the boundary of a boundary is zero, which implies that boundaries in 3D are always closed surfaces, which means that the index theorem cannot be applied anymore. It has already been shown that in 3D, critical boundaries, those hosting rotation, have a torus shape because the topological charge density is divergenceless (similar to magnetic field lines) [8]. The filaments you mentioned are an example of this; in this case, the phase defect is a tube of functional conduction block. The I, U, or O-shapes you mentioned have the same shape as a torus (in mathematical terms, they are homologous). We showed that in 3D, re-entry circuits are critical handle loops, contained in the homology basis. Importantly, these handle loops are not boundary loops, because they do not bound phase defect surfaces, so they do not enter Eqn 5.

   To summarize, the index theorem holds for all compact surfaces that are used to describe 2D and 3D data. However, in 3D, all rotational activity is captured on closed surfaces, so the index theorem is not applicable anymore. Instead, the divergencelessness of the topological charge density provides a new topological perspective, as it restricts rotational centers to a torus topology.

   As this part is out of scope of the current paper, we have added the following text to the limitations and future prospects section:

   "Finally, the methods in this paper are only applicable for two-dimensional surfaces with boundaries, which are topologically equivalent to n-holed spheres. We do not handle more complex geometries present with bi-atrial geometries or whereby the geometry of the ventricles includes wall thickness for example. However, we are currently working on a novel topological framework that generalizes this work for arbitrary topological surfaces. In this work, boundaries are redefined as the two-dimensional closed

surfaces that bound phase defect volumes, contrary to the one-dimensional loops that bound phase defect surfaces in this work. Preliminary results show that from these boundaries, the extraction of critical re-entry circuits in bi-atrial re-entry and ventricular tachycardia becomes possible."

2. *How would scroll waves in the septum affect interpretation on the epicardium. In the example given, the artificial boundary cuts out the septal influence so this is not an issue, but if the heart were rotated 90 degrees about the long axis, the septum wold be in the center of view.*

   answer: We assume that the reviewer is referring to O-shaped filaments in the septum between the left and right atrium, which could arise due to the thickness of the septum? In this case, the epicardium and endocardium of the left and right atrium will show focal activity, while the 3D rotation would be hidden. We illustrated this in our previous work, whereby we studied VT [17]. In the current study, we assume that there is no 3D reentry possible, as we assume we can approximate the left atrium and the right atrium as a sphere with no thickness. We refer to the limitations highlighted in the reviewers' question 1.

2 REVIEWER 2

1. *I thank the authors for presenting a practical and elegant potential solution to phase defects during phase mapping. There is a clear demonstration of how the extended method can make phase maps more interpretable and reliable in a range of relevant scenarios. My main concern is with the selection of the circular phase difference threshold $|\Delta\phi|$ (I will call it CPDT) used to remove phase defects. My reading is that, once the phase map is constructed, the method for detecting and removing phase defects effectively hinges on the choice of CPDT. It was briefly mentioned that the CPDT must be carefully selected as to not misidentify defects, such as identifying very slow conduction as block. There was no expansion on exactly how these were chosen for the study nor how one might go about deciding this threshold for different data. They seem to have been hand-selected based on manually reviewing the data, implying an assumption or prior knowledge of areas containing defects, especially in the case of functional block. Please expand this section of Methods to both describe exactly how you chose the thresholds in this study and provide a principled approach to selecting thresholds for new data sets. I appreciate an 'optimal' way of identifying and removing defects would use more than a single threshold parameter, but given that only one parameter is presented, the manuscript is currently lacking in explanation and exploration of it.*

   answer: We thank the reviewer for raising this important point that was not explained well enough in the manuscript. We are currently writing a paper that treats the optimization of this parameter in detail. Specifically, we use conduction block regions that were manually annotated by a clinician to find the optimal CPDT.

   For the present work, we have specified that values of the thresholds are hand-selected and stated in the limitations that we cannot guarantee that these values are optimal. We argued that the 'optimal' threshold would be the lowest values such that the only the phase defect is cut out.

   "First, the method used to detect phase defects in this study, relying on one parameter, is relatively basic and intended primarily for demonstration purposes. Because the thresholds were hand-selected, there is no guarantee that the chosen values are optimal. In this context, we define the "optimal" threshold as the lowest value for which only the phase defect is removed. To make phase mapping more reliable and robust for interpreting realistic data, more sophisticated approaches relying on more features of the electrogram for phase defect detection should be explored. For example, features such as low voltage, mid-diastolic potentials, after-depolarizations, double potentials, fractionation, and other electrogram characteristics associated with decreased cardiac conduction could provide more robust indicators of phase defects and are valuable future research topics. Furthermore, methods that leverage machine learning could also be useful to identify phase defects as an input to phase mapping, without replacing the phase mapping analysis itself."

2. *Just as a check on my understanding, would a CPDT of 2π effectively reduce the extended method to the naïve case, as the maximal circular difference would be permitted thus no defects are removed?*

answer: Almost, the phases and modulated phase differences live in the domain $[-\pi, \pi[$, therefore it is a CPDT of π that will ignore all functional blocks. However, we should note that this is not completely the same as the naive method since the phase index around structural block (panel 2 in Figure 2) and boundaries (panel 3 in Figure 2) will still be calculated in the extended method, while it will be ignored in the naive method.

In addition, we have added some notebooks to our code repository where you can play around with different values. `https://gitlab.com/opendgm/paper-phase-defects`

3. *How is the outer boundary defined on a complex 3D geometry – is it the largest geodesic that traverses the entirety of the mesh? Relatedly, all the examples presented are of a "single boundary" domain; how would the outer boundary be defined for multiple geometries that "go together" like you might see in a bi-atrial circuit (where the LA and RA meshes would both need consideration)? Could the phase map be continuous through the septum, or would you need to interpret 2 separate phase maps? Clinically mapped meshes like in CARTO are usually multiple separate surface meshes registered in the same space, so how is the outer boundary defined – separately for each mesh, or together?*

   answer: Thank you for raising this interesting point. When you say "outer boundary" we assume you are referring to the boundary of the flat tissue presented in Figures 5-8. It is important to understand that the shape of this tissue is equivalent to a spherical surface with a hole (see Fig 1). The "largest geodesic" you suggested does not fit here, as geodesics are defined as the shortest path between two points, so if the path were to close in on itself to form a loop, it would not be a geodesic.

   The clinical endocardial CARTO map of the left atrium we analyzed has the shape of a sphere without holes. This is a single map of the endocardium, not the combination of multiple maps as you suggested. Since the shape of the map is a sphere, there is no outer boundary. It is only by removing the tissue that has bad signals (near the valves), that holes are generated. In the left atrial endocardium example, three such holes are commonly annotated (LPV, RPV, MV), meaning the end result is a sphere with three holes. The outer boundary of such a map would then be the combination of the three disjoint loops that surround the holes. In case multiple maps go together, such as the bi-atrial map, the total map would merge the maps of the LA and RA endocardium via the septum via N connections, so the resulting map will contain no boundary. After removing the valves, the end result would be two spheres with 3 holes each that are connected via N connections.

   However, the case of a bi-atrial circuit is very interesting as it highlights the flaws in our n-holed sphere model. In our model, rotation is only possible around boundaries, but the bi-atrial circuit does not travel around a valve; it travels from the left to the right atrium via connections in the septum. The bi-atrial circuit is therefore not a boundary. In our new topological framework, we model the bi-atrial surface as a genus-g surface (Fig 2), where $g = (6-1) + (N-1) = N + 4$ denotes the number of topological holes. In this model, we allow rotations along loops on this surface that can't be shrunk down to a point or smoothed away. These kinds of loops are captured mathematically by something called the first homology group, a standard tool in topology for classifying such loops.

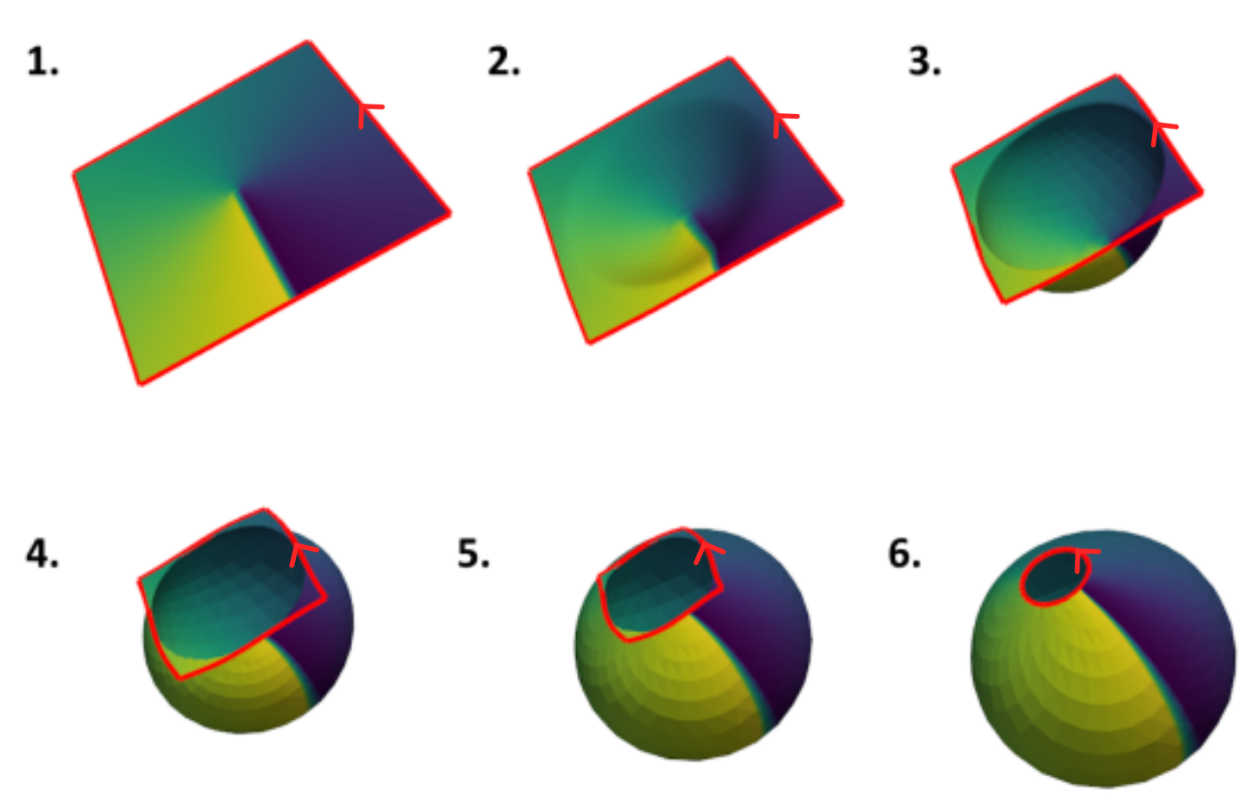

Figure 1: Deformation of a flat tissue to a one-holed sphere

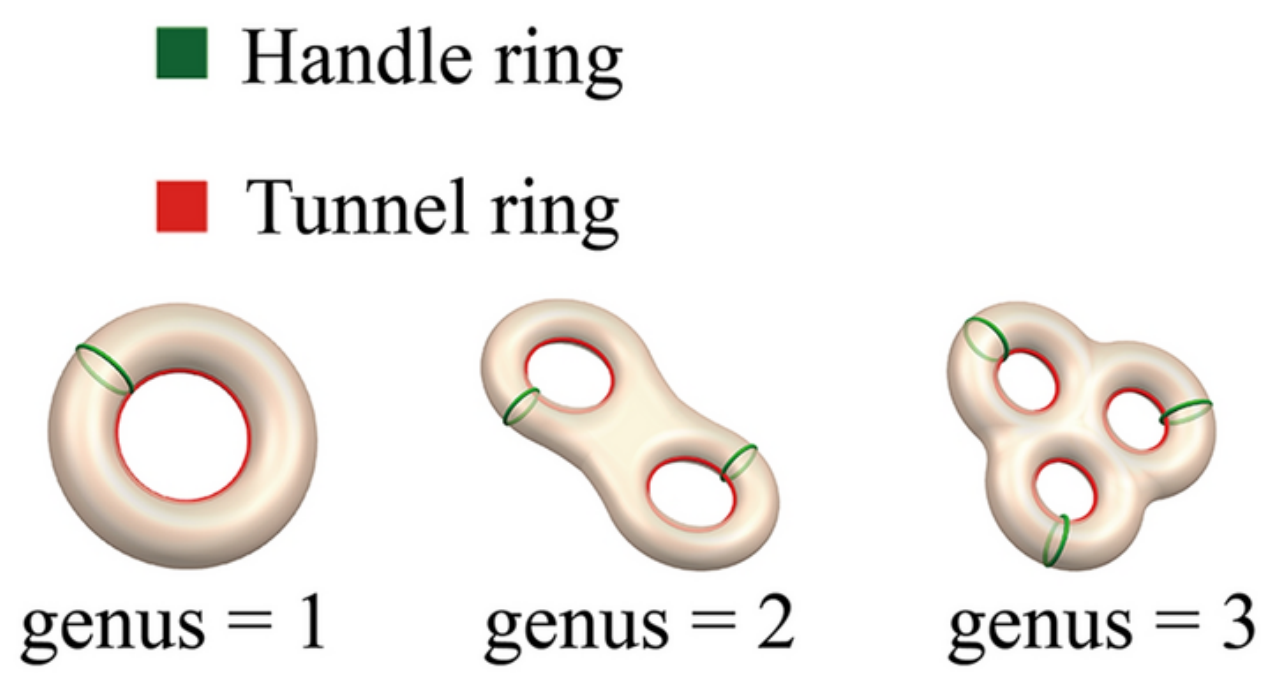

Figure 2: Loops of the first homology group (handles and tunnels) on genus-g surfaces

4. *Even after flicking through the manuscript referenced for it being "convention", it is not immediately obvious to me why the outer edge is integrated in the opposite direction (e.g in Fig 5) – from reading your Methods it seems to be to distinguish it from the inner edges – is that correct?*

   answer: Yes, this is indeed a consequence of the surface topology. To illustrate this, consider Figure 1. Although the surface is represented as a flat sheet, it is topologically equivalent to a sphere containing an anatomical hole. The integration convention uses the right-hand rule (Figure 3), where the right-hand thumb is the normal of the surface, and the other fingers follow the integration path. For the outer boundary on Figure 1, the index is computed by traversing the boundary clockwise, such that the enclosed tissue remains on the left-hand side of the integration path.

   Following the same convention, for the inner boundary around the center of the tissue in panel 1 of Figure 1, the contour is traversed counter-clockwise, as the normal of the tissue center points downwards. However, for the outer boundary of the flattened surface, the equivalent contour on the closed surface is viewed from the opposite side, which reverses the orientation. Consequently, the index must be computed in the counter-clockwise direction along the outer boundary, and clockwise along the inner boundary.

   We agree that this point may not be immediately intuitive and have emphasized that we follow the convention of previous work in the methods section:

   "The direction of integration follows the approach of [3], and is indicated in the figure."

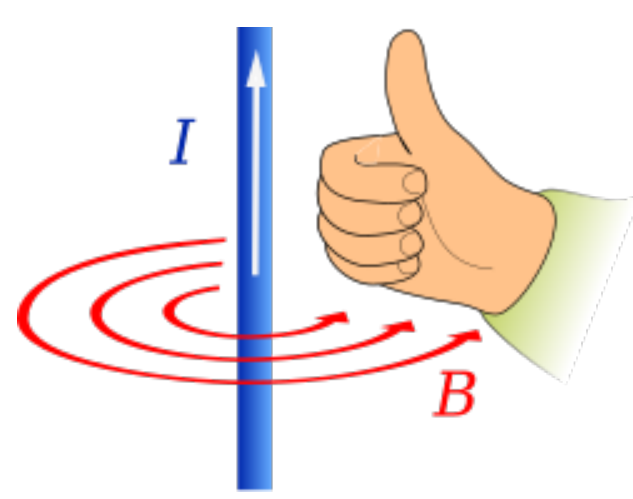

Figure 3: The right hand rule

5. *Outer edge absolute phase index > 1 due to multiple wavefronts is interesting; does it also imply the wavefronts are travelling in opposing directions relative to the direction of integration of the outer edge?*

   answer: The absolute value of the phase index does not determine whether the wavefronts are travelling in the same direction or opposite to the integration direction. It is the sign of the phase index that determines this. A positive phase index indicates that the wavefronts travel in the same direction as the integration direction, while a negative phase index indicates the wavefront and integration direction are opposite.

   Additionally, an absolute phase index of 2 means that the net difference between opposing wavefronts is two. This scenario can occur if there are two wavefronts traveling in one direction and zero in the

opposite, three versus one, four versus two, etc.

6. *How is conduction block due to wavefront collisions treated – would the defects effectively trace a path of defects caused by the wavefront collisions, visualising a kind of "isthmus" where the boundaries creating the isthmus are mesh holes that have been removed?*

   answer: We thank the reviewer for this interesting question. The answer is no as wavefront collisions are continuous in the phasefield, and are therefore not considered defects. However, they are not continuous in the velocity vector field, but this is not a requirement for the path of the phase index calculation.

7. *The CARTO meshes I work with are usually 1mm resolution, but yours is 0.4mm – was this refined and re-interpolated, or was it taken directly from CARTO?*

   answer: The mesh was obtained directly from the CARTO export without any modifications. We collaborate with AZ Sint-Jan, which provided the clinical data used in this study.

   This was emphasized in the sentence in the methods section:
   "Mean electrode–mesh distance < 4 mm"

8. *How long do the extended phase maps take to compute? Is it feasible to compute the phase map, or to recompute with different parameters e.g updating CPDT, in near-real time? Must they be computed after an entire map has been collected e.g a full chamber LAT map?*

   answer: The last version takes under 0.1s for 1 timestep such as for atrial tachycardia, with a mesh size as described in the methods section. For simulations with multiple timesteps, it would take around 14 sec for 400 timesteps in a mesh with 569467 points on a regular laptop (intel i7 12700H). While one can indeed analyze part of a map, similar to the flat 2D examples in the paper, one must always account for the boundary around the tissue segment.

9. *I think re-organising the current Methods subsections to have the following structure makes for a more logical reading order: phase mapping fundamentals (Phase mapping); phase defects and the extended phase mapping approach to remedy them; simulation/experimental/clinical setups used for demonstration (Study cases).*

   answer: Thank you for the feedback. We have rearranged the method section as you suggested. This rearrangement is not included in the tracked changes as it would clutter the document.

10. *The results section is wanting for a graphical representation of the comparison between naive and extended phase mapping. A list of categories to show include but are not limited to: number of phase defects (removed/remaining in the extended case, if there are phase defects remaining above the threshold that were not removed), number of true rotors missed, number of false rotors detected. A stacked bar plot with the bars colour-coded with "simulation", "experimental", "clinical" might work as a good visual representation, although this is just a suggestion, and you may find a different visual representation has more explanatory power.*

    answer:

    Thank you for your suggestion, we also feel that additional figures would benefit the results section. Based on your suggestions, we chose to depict the number of positive and negative rotations, in addition to the phase index sum of all rotations for the naive and extended method. We refrained from calculating metrics such as true or false positives, as we did not manually label the ground-truth location of rotational centers and phase defects. Such a time consuming analysis could in principle be done, but we believe this does enforce the main idea of the paper enough to be worth the effort.

    We have added these figures to the results section and slightly modified the text, which can be seen in the updated document. We also added the following text to the discussion:

    "Taken together, these results highlight a fundamental limitation of the naive approach: it neither reliably detects rotational activity nor adheres to the index theorem, which requires the sum of phase singularity indices over a compact surface to equal zero. Violations of this constraint are observed across all datasets, reflecting the ill-defined nature of the phase index in the presence of phase defects.

The extended approach, by computing the phase index around rather than through phase defects, restores theoretical consistency: the index theorem is satisfied in all cases, and the detected singularities carry genuine interpretive value as markers of rotational activity."

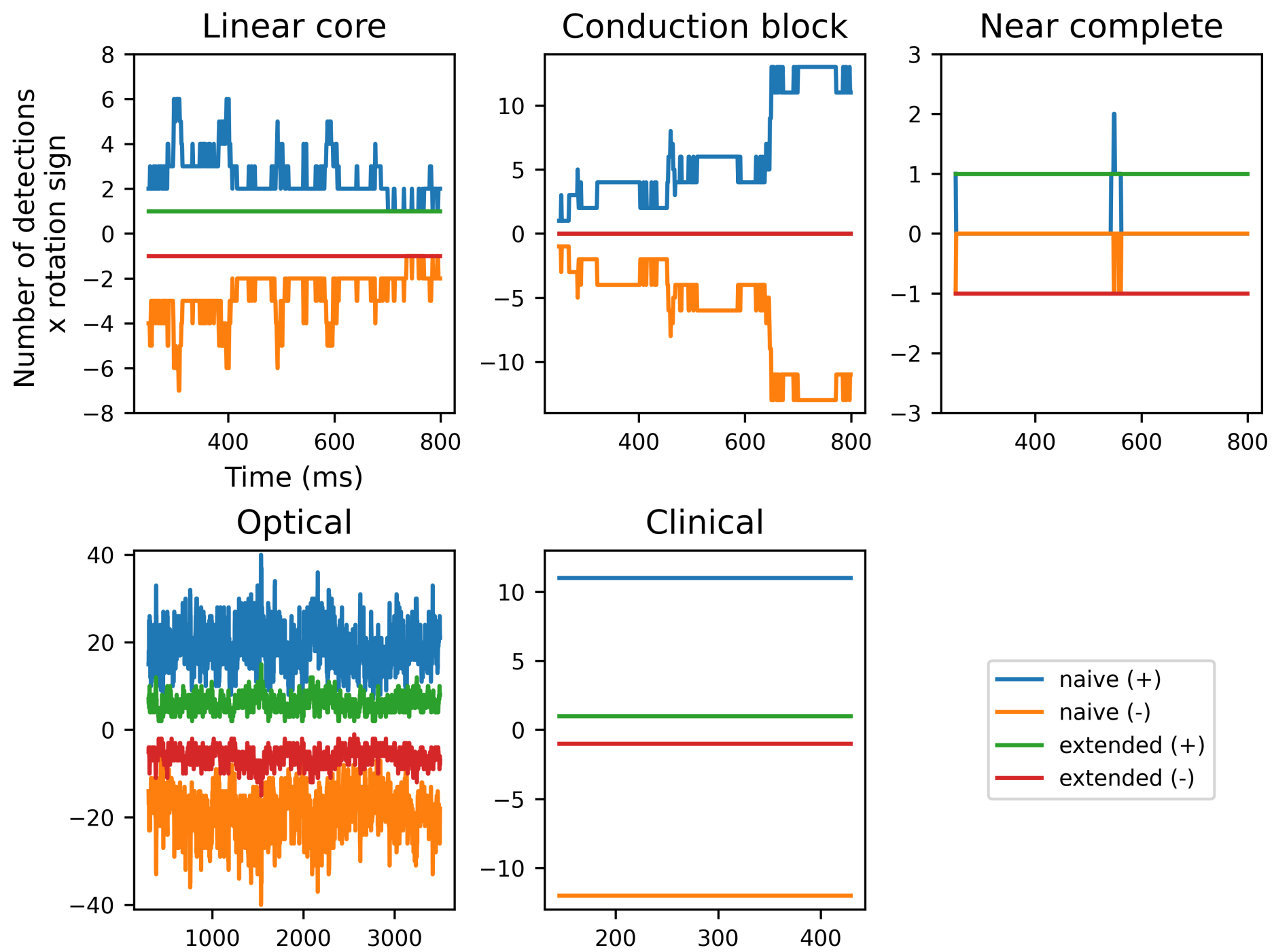

Figure 4: Number of detected phase singularities (naive method), and critical phase defects (extended method) over time for all five datasets. The naive approach has a higher number of detections and greater variability over time compared to the extended approach.

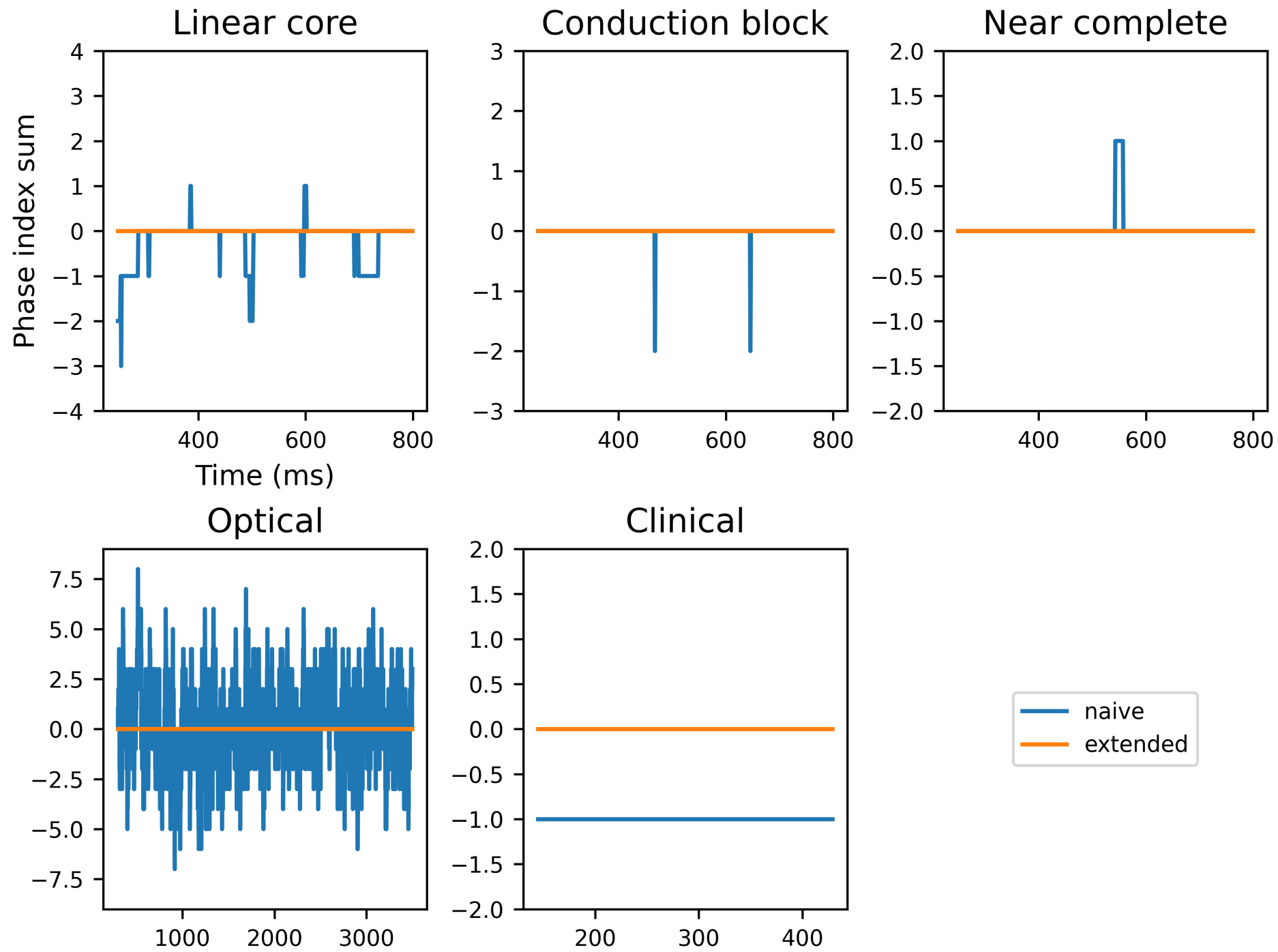

Figure 5: Phase index sum of phase singularities (naive method), and critical phase defects (extended method) over time for all five datasets. The naive approach violates the index theorem, while the extended approach adheres to the index theorem for all time frames but one.

11. *Figures 6-9 could be combined into one figure – there is a lot of repetition in the captions, and the figure layout and colours are nice and consistent, so it makes sense to combine them into a single figure and put the snapshot time in the row.*

    answer: We thank the reviewer for the suggestion, and we have merged the figures 6-8 into fig 6, but left Fig. 9 separate so that we could show more snapshots.

12. *Out of interest, what would happen if there was a phase defect within the midmyocardium? The atrial free wall is thin but what about thicker areas like Bachmann's bundle which may participate in bi-atrial circuits? For a volumetric mesh, could you bound the defects by a surface instead of a curve to identify phase defects and remove volumes? I appreciate this can get complicated and is almost certainly out of scope for the manuscript*

    answer: We commend your insight on the topic, your suggestion of bounding the defects by a surface instead of a curve is exactly the foundation of our ongoing research on rotations in three dimensions. Regarding the application for volumetric meshes, we refer to both questions of reviewer 1. For the bi-atrial circuits, we refer to your question 4.

    Added text in the manuscript:

    "Finally, the methods in this paper are only applicable for two-dimensional surfaces with boundaries, which are topologically equivalent to n-holed spheres. We do not handle more complex geometries present with bi-atrial geometries or whereby the geometry of the ventricles includes wall thickness for example. However, we are currently working on a novel topological framework that generalizes this work for arbitrary topological surfaces. In this work, boundaries are redefined as the two-dimensional closed surfaces that bound phase defect volumes, contrary to the one-dimensional loops that bound phase defect surfaces in this work. Preliminary results show that from these boundaries, the extraction of critical re-entry circuits in bi-atrial re-entry and ventricular tachycardia becomes possible."

3 REVIEWER 3

1. *My main concern is that the method is demonstrated on only a few datasets. Especially, the primary potential use case, clinical data of atrial tachycardia or data with multiple near-complete rotations, is shown for only one case and should be explored more carefully using other examples. In particular, I am curious how noise affects the analysis.*

   answer: We thank the reviewer for this important and insightful remark.

   To address the first point regarding the dataset size, we agree that validation on a larger clinical cohort is essential. In fact, we are currently preparing a comprehensive follow-up manuscript that applies this extended phase mapping method to a clinical dataset of over 200 AT cases. Our preliminary results on this large cohort demonstrate that the method can automatically identify critical boundaries without manual intervention. However, because these clinical findings are highly extensive, including them here would compromise the focus and readability of the current paper.

   The primary objective of the present manuscript is fundamentally theoretical: to demonstrate that the standard implementation of phase mapping, a method used for over 30 years, contains a core mathematical error. We believe establishing this mathematical correction is a necessary, standalone prerequisite before deploying the method on a larger scale. As we noted in the limitations section:

   "The primary aim of this work is to highlight the challenges that arise when phase defects are present and to demonstrate the potential benefits of explicitly addressing them during phase mapping analysis. As such, this study should not be viewed as a detailed statistical analysis on a large dataset investigating phase defect detection algorithms and robustness."

   However, we also agree that it is interesting to test the impact of noise. Following your excellent suggestion, we have performed an additional analysis to analyze how noise affects the robustness of our method. We have added the following text to the limitations:

   "However, we have included some preliminary results in the appendix (Fig 6, 7), illustrating that Gaussian noise on the phase map manifests itself as phase defects for our implementation relying on phase differences. For noise levels of above $\sigma = 0.2\pi$, the naive method (threshold = π) breaks down as multiple false positive phase singularities are found. The extended approach instead converts false positives into phase defects by lowering the phase difference threshold."

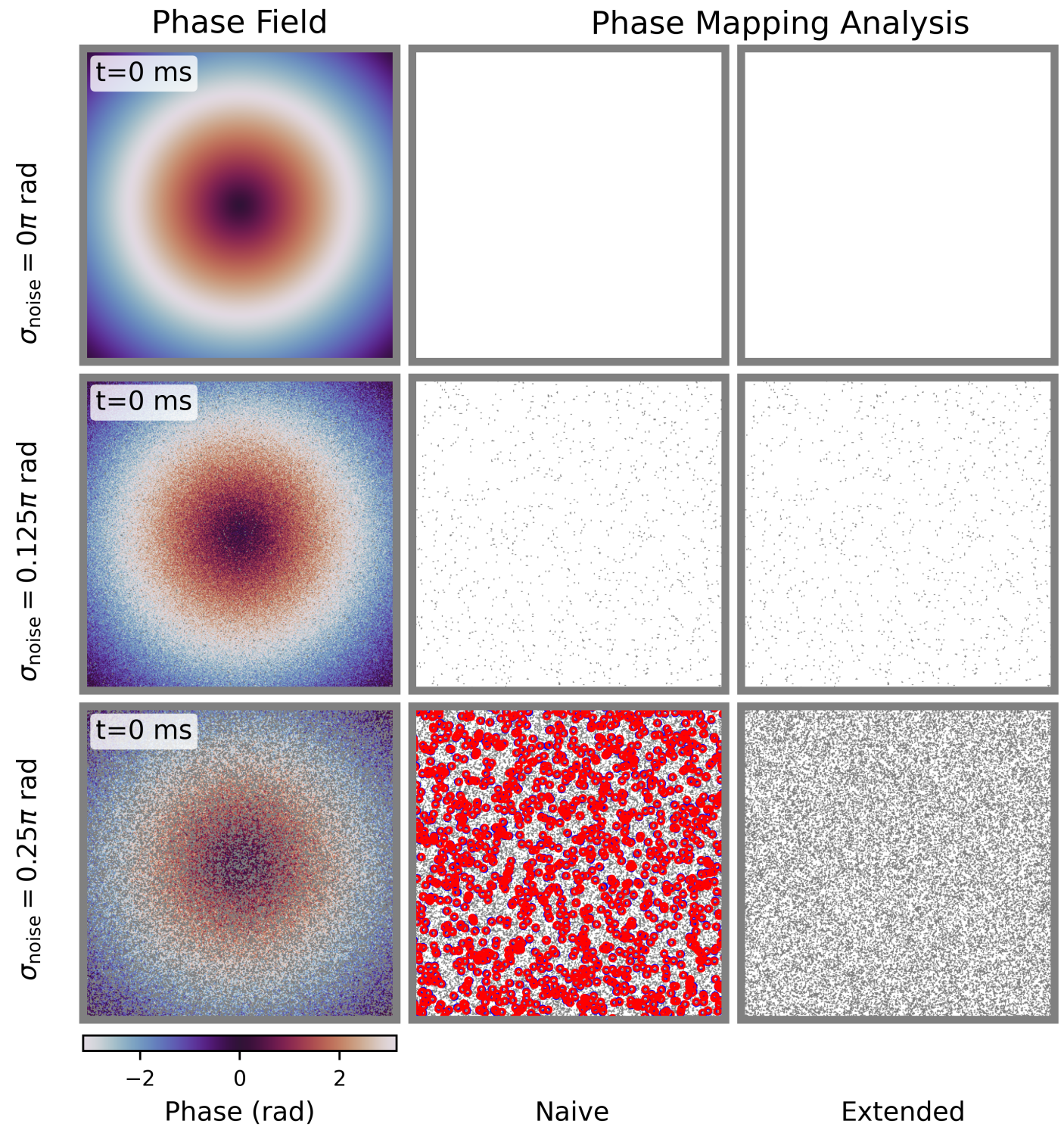

Figure 6: Illustration of naive and extended phase mapping for an S1 simulation on a 400x400 grid with increasing levels of Gaussian noise. The naive method breaks down for noise with a standard deviation of $\sigma_{\text{noise}} = 0.12\pi$ as multiple false positive detections are found. The extended approach (with a phase difference threshold of 0.5π) is robust against all levels of noise as no critical phase defects are found.

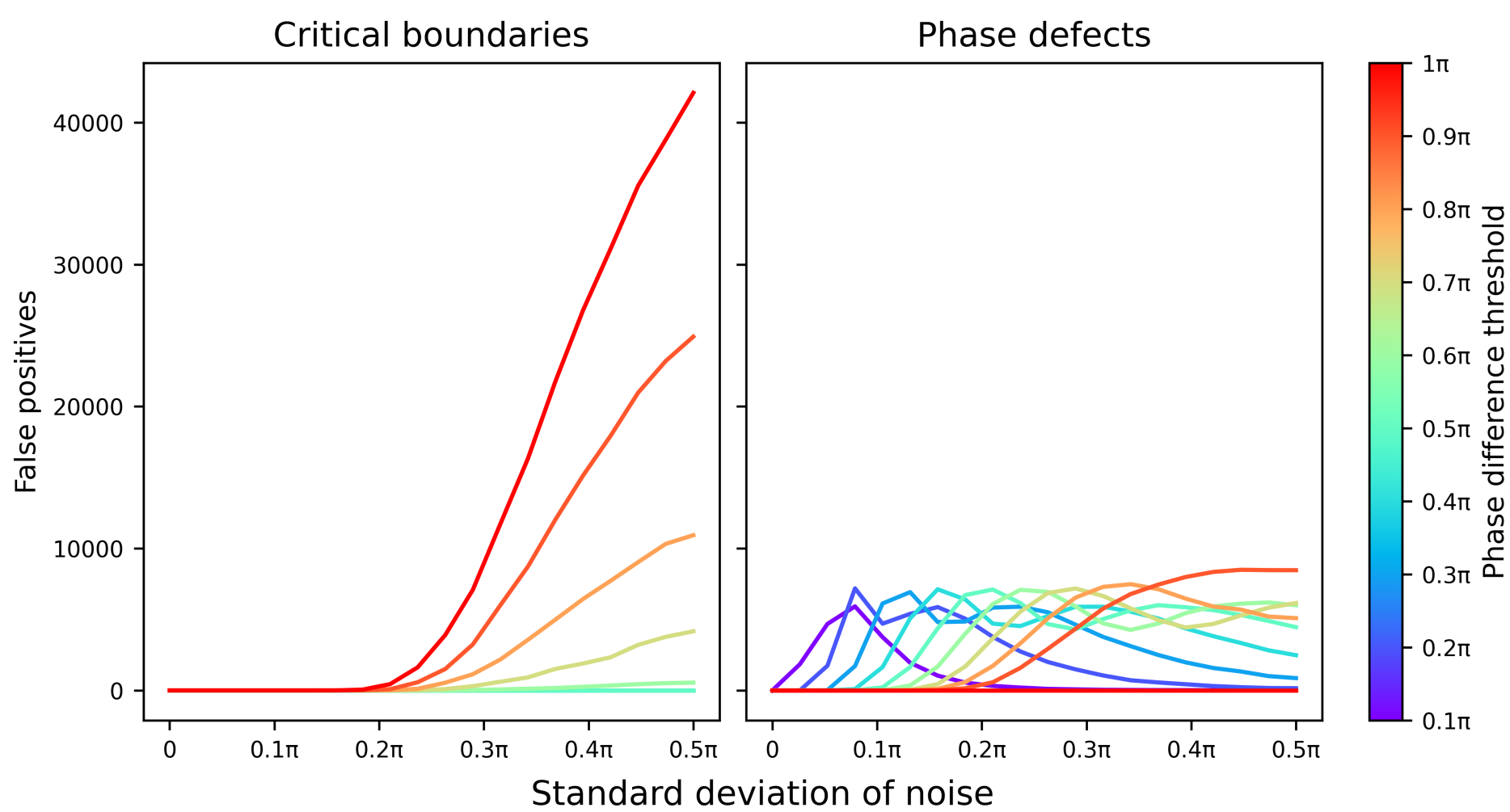

Figure 7: Illustration of the effect of noise and the phase difference threshold on the number of false positive detections in an S1 simulation. Note that a phase difference threshold of π closely resembles the naive approach, with the inclusion of the boundary phase defect. Decreasing the phase difference threshold converts the false positive critical boundaries to false positive phase defects. The naive approach breaks down for noise levels above 0.2π, while the extended approach contains no false positive critical boundaries for all levels of noise for a phase difference threshold below 0.5π

Furthermore, we refer to the answer to question 11 of reviewer 2, where we included a more in-depth analysis of our data. Despite our limited dataset, we believe this additional analysis further supports our claims.

2. *With ventricular fibrillation data, the main improvement seems to be simply avoiding false-positive detections. However, as the authors note, such false-positive detections can also be removed using previously published techniques. Considering this, what is the added benefit of the method for ventricular fibrillation? Is there something new we can learn about ventricular fibrillation with this method?*

   answer:

   We thank the reviewer for this excellent question. We would like to emphasize that conventional approaches for removing false-positive phase singularities are generally not derived from the underlying mathematics of phase mapping. Instead, they assume that the phase field is continuous and subsequently rely on heuristic post-processing steps, such as clustering detected phase singularities using arbitrary parameters (e.g., clustering radius, minimum cluster size, or minimum number of rotations). Machine-learning approaches similarly introduce large numbers of parameters that are not always physiologically interpretable.

   In contrast, our approach does not cluster or filter detections. It is motivated directly by the mathematics of the phase index, which is only well defined when phase continuity is properly accounted for. We therefore shift the focus from post-processing phase singularities to directly and accurately identifying phase defects without any need for additional post-processing. Our central claim is that, once discontinuities are explicitly handled, every detection corresponds to a complete or near-complete rotation of electrical activity around a phase defect, eliminating both false-positive and false-negative detections within the framework of our methodology.

   We further argue that conventional phase-mapping approaches combined with clustering implicitly assume phase continuity. When this assumption is violated, the phase index can be computed incorrectly, leading to both spurious detections and missed phase singularities. In the present work, phase continuity is modeled using a simple phase-difference threshold. While this provides a practical solution, more sophisticated methods for characterizing continuity should be investigated in future work.

   Finally, our method not only reduces false-positive detections but also avoids false negatives that commonly occur near regions where the phase is undefined, such as scar tissue or anatomical boundaries in ventricular fibrillation data.

   We have added the following text to the discussion:

   "Beyond theoretical consistency, the extended approach offers a conceptually distinct alternative to conventional false-positive suppression. Existing post-processing strategies such as phase singularity clustering or minimum-duration filters implicitly assume phase continuity and rely on heuristic parameters that lack direct physiological interpretation. The extended approach instead grounds false-positive suppression in the mathematics of the phase index: by explicitly modeling phase discontinuities, every detection corresponds to a complete or near-complete rotation around a phase defect, eliminating the need for arbitrary post-processing. Additionally, the extended approach also avoids the false negatives that the naive approach introduces near regions of undefined phase, such as scar tissue or anatomical boundaries. While the phase-difference threshold used here provides a practical means of identifying discontinuities, more sophisticated characterizations of phase continuity remain an avenue for future work."

3. *In the conclusion, the authors write: "These findings challenge the classical view that rotors or functional reentry in cardiac arrhythmia are sustained by physical rotation around singular points." The term "challenge" is not appropriate in my view as the authors do not fully demonstrate their technique with 2D observations of scroll waves and appear to miss the point that the concept of a phase singularity was not invented to characterize rotor dynamics in detail, but rather to simplify the complexity of wave patterns measured across the surface of the ventricles during ventricular fibrillation. While the proposed extensions can be useful in some instances, other researchers may not necessarily want to adopt these concepts for their data, as a singular point is easier to track and visualize over time than lines and loops.*

answer: We agree that "challenge" was not the right word. We also agree that phase singularities were introduced as a practical simplification, not to characterize rotor dynamics in detail. But if the study of wave patterns relies on singularity detection, the correctness of that detection matters regardless. The standard implementation produces ill-defined results whenever the integration path crosses a phase discontinuity, leading to both false positive and false negative detections. We are convinced that they cannot be resolved in a consistent manner by post-processing. Our strongest argument for this is that conventional singularity detection is blind to rotational activity around large anatomical or functional obstacles, especially near-complete rotations. A wave pattern that has been shown to have clinical relevance [6, 13, 15]. Our approach recovers them directly, together with reentries and singular points. Our goal is not to present a new method or to convince the reader to abandon singular points. As stated in the limitations, we encourage research groups to account for phase defects. How they choose to do so is left to them. To better reflect this scope, we have changed the title to :

"Going around phase defects: addressing a limitation of phase singularity detection"

In the same spirit, the last sentence of the abstract is changed to:

"Therefore, we propose that explicitly accounting for phase defects, rather than tracking singular points alone, gives a more complete and consistent characterization of rotational dynamics in cardiac tissue."

and the conclusion now reads:

"We demonstrated the influence of phase defects on the detection of phase singularities using a naive phase mapping implementation across five case studies encompassing simulated, experimental, and clinical data. We found that the naive method produces both false positive and false negative detections, making the interpretation of wave patterns inconsistent and more convoluted. In contrast, the extended approach provides consistent results and interprets the drivers of rotation as phase defects with a non-zero phase index. The findings highlight the limitations of using singular points to characterize wave patterns, particularly in clinical settings where near-complete rotations around large obstacles are critical targets for ablation therapy. Instead, we propose that characterizing arrhythmia drivers as complete or near-complete rotation around phase defects provides a more complete picture than singular points alone. Rather than promoting our specific implementation, we emphasize the necessity of explicitly addressing phase defects in phase mapping analyses. We hope this work motivates further research into the role of phase defects and encourages their integration into analytical workflows. "

4. *Numerous studies have tracked rotor cores and characterized drift and rotor core trajectories using phase singularities. Different spiral wave regimes and model parameters were associated with different meandering patterns. Such patterns take the shape of an epitrochoid or hypotrochoid with inward or outward petals. The present study is lacking such an analysis. The authors should either add a paragraph discussing how their framework would impact such studies and forms of analysis, or present new data characterizing meandering rotors while providing a succinct and meaningful analysis of such patterns that provides new insights into spiral wave dynamics.*

   answer: A discussion of meandering patterns are indeed lacking and therefore, we have added a paragraph in the discussion:

   "Beyond the scope of this work, previous studies have characterized meandering patterns of spiral waves, often described as epitrochoid or hypotrochoid trajectories [19, 1]. While this work centers around phase defects and leans into the hypothesis that singular points are not representative for rotational activity, it does not necessarily invalidate such studies. However, it would be more suitable to use wavefront endpoints instead of singular points for such studies. Assuming these endpoints are always attached to a phase defect [2, 16], studying how phase defects evolve over time offers a new way for characterizing such patterns. For example, the simulation of a linear rotor core (see S1 Video) shows an elongated defect around which the wavefront rotates. The phase defect itself also rotates, but at a slower rate than the wavefront rotating around it. In this case the meandering pattern can be described by the size and rotational speed of the phase defect, and the rotational speed of the wavefront endpoint around the defect. These variables should in principle be sufficient to describe epitrochoid or hypotrochoid trajectories [1]."

5. *The study does not account for 3D dynamics. If the authors aim to make a meaningful contribution to the analysis of ventricular fibrillation, they should demonstrate their technique with a 2D/3D analysis by comparing the wave patterns on the surface versus within the volume.*

   answer: We kept the scope of this work to phase mapping analysis in 2D. However, the research of this subject in 3D is already in progress, with the basis paper available in Van Den Abeele et al.[17]. As a 3D analysis is out of scope of the current paper, we have added the following text to the limitations and future prospects section:

   "Finally, the methods in this paper are only applicable for two-dimensional surfaces with boundaries, which are topologically equivalent to n-holed spheres. We do not handle more complex geometries present with bi-atrial geometries or whereby the geometry of the ventricles includes wall thickness for example. However, we are currently working on a novel topological framework that generalizes this work for arbitrary topological surfaces. In this work, boundaries are redefined as the two-dimensional closed surfaces that bound phase defect volumes, contrary to the one-dimensional loops that bound phase defect surfaces in this work. Preliminary results show that from these boundaries, the extraction of critical re-entry circuits in bi-atrial re-entry and ventricular tachycardia becomes possible."

6. *The main finding of the paper, in my view, is shown in Fig. 10, in which the phase defects are displayed as lines that surround the large holes, while short-lived phase singularities are ignored. This figure needs more details. A few other views and other snapshots of different phases of the recording would be helpful to better assess the performance. The caption should detail what the red and blue lines mean physiologically. For example, do they correspond to a clockwise or counter-clockwise rotating activation?*

   answer: We followed the suggestions of the reviewer and we have added three perspectives (AP, LAO and PA) and specified the rotational direction in the caption (see Fig. 8).The singularities and phase defects are static so adding more snapshots would not give additional information. We would also like to refer to our Zenodo repository where we added a video that displays the analysis for all the time frames.

7. *In Fig. 10, the LAT and phase maps are discontinuous. Are they supposed to be like that? If not, then this should be improved and be displayed without discontinuities.*

   answer: It is expected. The discontinuities are caused by bad interpolation of the LAT values in regions with lots of scar tissue. In this case the discontinuity is caused by a previous ablation line between LPV and MV and scar tissue nearby the appendix.

8. *Also, please add annotations of the anatomical features and defects.*

   answer: Anatomical features and defects are annotated in gray, and we have labeled the anatomical structures of clinical activation map in both Fig. 5 and Fig. 8.

9. *Fig. 6 would benefit from additional snapshots showing the rotor over several time steps. Please create additional subpanels, which show the rotor pattern at different time steps as in the corresponding video. Otherwise, statements in the text, such as "The naive approach identifies one or multiple phase singularities of both positive and negative phase index values at the central defect per time frame." (line 270ff) do not match with the figure. Moreover, Fig. 6 would benefit from annotations, e.g. arrows pointing at the different components, index values, etc.. Also, the phase singularities and defect lines could be drawn a bit thicker.*

   answer: We followed the suggestion of reviewer 1 (question 12) to merge the figures of the simulations. Therefore, showing more time steps would make the figure to big. Instead, we have removed the statements about the time evolution. We have added a video to our Zenodo repository that displays the analysis for all the time frames Also, our code repository contains a notebook `notebook/analyze_simulations.py` that shows an interactive slider of the analysis so that readers can explore the different time steps by themselves. For all figures, we added the direction of the wavefront and made the lines of the wavefronts, defects and singularities thicker. We hope that these revisions address the reviewer's concerns.

10. *In Fig. 6, why are there sometimes two blue lines at the rotor core and what do they mean?*

    answer: We have added a few sentences in the limitations to explain the origin of such branched structures.

    "Beyond the choice of activation threshold, both the Hilbert Transform and sawtooth phase definitions break down near phase defects. This can be seen in Fig. 6 where the defect in the linear core simulation has branched structure. The branches at the rotor core are an artifact of phase mapping, where linear interpolation between sequential LATs breaks down near phase defects due to aperiodicity. Since the artifact mimics the signature of a true phase defect, it can lead to false-positive detections: phase singularities in the naive approach and incorrectly shaped phase defects in the extended approach. It should therefore be interpreted with caution."

11. *In Fig. 6, what meaning does the outer boundary phase defect line have? What practical relevance does it have? In the discussion the authors write that "... the linear rotor core and a single static critical phase defect denoting the outer edge of the tissue. This is interpreted as paired reentry, with a central semi-stable complete rotation and a stable near-complete rotation at the boundary." What is the physiological relevance of this sentence? Please clarify.*

    answer: Rotation around the outer boundary on a flat tissue is topologically equivalent to rotation around a hole such as the mitral valve in clinical atrial tachycardia (see Fig 1). The deformation of a flat tissue to a sphere with a removed disk has been described in question 6 of reviewer 2. With this in mind, the situation on the flat surface is equivalent to paired rotation around a functional phase defect and a hole on a sphere. This corresponds to a geometry with 2 boundaries, for which the re-entry circuit is technically a single loop, which can occur in clinical AT with re-entry around the MV and roof. The two loops around the boundaries in this case are not independent; they can be deformed into one another without cutting them (homologous). In case more than 2 boundaries are present, we call the re-entry circuit a double loop, which can occur in clinical AT when the roof is not ablated. To generalize, we find it useful to classify all re-entry circuits as paired re-entry (always double loop).

12. *In Fig. 7, please indicate the propagation direction with an arrow.*

    answer: We thank the reviewer for this suggestion. We have indicated the direction in the new figures, see Figures 3-6.

13. *In lines 285-286, it is stated that "the extended approach finds an average of zero critical phase defects and seven non-critical phase defects per time frame". This is not obvious from the figure. Please modify the figure so that individual phase defect detections can be seen over time. What do these numbers reveal?*

    answer: We thank the reviewer for this comment; this was indeed not clear from the figures provided. As such, we have added a figure that illustrates the time evolution (see Fig 4). Furthermore, we would like to remind the reviewer that we already provided videos in our Zenodo Repository and a notebook showing an interactive slider in case readers want to explore the different time frames.

14. *It is not clear to me what the added value of the new method is when applied to ventricular fibrillation. In Fig. 9, it appears that the only difference is that the phase singularities are replaced by little rings and that short-lived phase singularities disappear. For the case of ventricular fibrillation, what can this method visualize that the naive method cannot?*

    answer:

    We assume that the reviewer feels that the extended approach does not provide a significantly different result than the naive approach for the ventricular fibrillation data based on the visual result. First, we would like to stress that there is in fact a significant difference, as many false positive detections of the naive implementation vanish in the extended approach. Furthermore, rotation around singular points is converted to rotation around two-dimensional surfaces.

    Second, we would like to emphasize that the difference between both methods lies deeper than purely the visual result. In ventricular optical mapping data of ventricular fibrillation, most rotational activity is functional re-entry. As such phase mapping is expected to perform well because phase singularities

supposedly model functional re-entry. Traditional analyses have already revealed that some phase singularities live longer than others, and they are often filtered according to their longevity to focus on the most important rotational drivers. In this paper, we claim such strategies are not robust, and try to clean up false detections that shouldn't be there in the first place. We claim that the false positives appear due to the violation of the continuous phase assumption that conventional implementations make. We propose an alternative solution that removes the false positives from appearing in the first place, instead of post-processing. This is done by shifting the main idea of rotor detection from rotation around singular points to rotation around two-dimensional phase defects. Using this strategy, it becomes clear that many supposed rotational centers detected by naive implementations vanish, as they are actually just wavefront endpoints attached to a functional conduction block. In essence, our method aggregates wavefront endpoints for all phase defects: functional conduction block, structural conduction block and tissue boundaries, resulting in a net rotation around said phase defects. This is conceptually different from rotation around a singular point.

We have included the following text to the discussion:

"For the optical mapping data, the naive approach implicitly assumes a continuous phase, causing wavefront endpoints attached to functional conduction blocks to appear as spurious rotational drivers. Our method shifts the paradigm from rotation around singular points to rotation around two-dimensional phase defects, inherently eliminating these false positives rather than filtering them in post-processing."

15. *The supplementary videos are not referenced correctly in the text.*

    answer: We thank the reviewer noticing. This issue has been fixed.

16. *The sentence at the beginning of the discussion, "This section interprets the results presented above, ... ", is redundant. The whole section could be more succinct.*

    answer: We have shortened the section as follows:

    "The three simulations in Fig 6 reveal a consistent failure of the naive approach: false positive phase singularities are detected whenever a functional conduction block is present. These false detections could arguably be filtered out by clustering [10, 11, 18] or by requiring a minimum duration for a singular point to persist [11]. However, the naive approach also misses near-complete rotations around large obstacles due to the undefined phase of scar tissue, and such near-complete rotations would still be discarded by a minimum-duration filter. Consequently, when an activation map contains multiple types of phase defects, no straightforward post-processing strategy exists to resolve these competing errors. The root cause, importantly, is not noise — since these simulations are noise-free — but rather that the phase index is fundamentally ill-defined in the presence of phase defects. The extended approach directly addresses both failure modes by computing the phase index around phase defects rather than through them. This eliminates false positives arising from functional conduction blocks while simultaneously recovering near-complete rotations, as demonstrated in the third simulation. The latter is of particular clinical relevance: recent studies have shown that near-complete rotation can play a critical role in guiding ablation strategies [6, 13, 15].

    The optical mapping and clinical data reinforce this picture. In both cases, singular points are consistently attached to phase defects, suggesting they reflect the presence of a defect rather than genuine rotational activity. For the optical mapping data, the naive approach implicitly assumes a continuous phase, causing wavefront endpoints attached to functional conduction blocks to appear as spurious rotational drivers. Our method shifts the paradigm from rotation around singular points to rotation around two-dimensional phase defects, inherently eliminating these false positives rather than filtering them in post-processing. For the clinical map, the naive approach fails to detect the reentry around the anterior scar and the near-complete rotation around the MV and LPV. The extended approach, by contrast, demonstrates the full interpretive value of phase mapping: false positives are filtered out, complete rotation is detected around the anterior scar, and near-complete rotation is identified around the MV and LPV.

    Taken together, these results highlight a fundamental limitation of the naive approach: it neither reliably detects rotational activity nor adheres to the index theorem, which requires the sum of phase singularity indices over a compact surface to equal zero. Violations of this constraint are observed

across all datasets, reflecting the ill-defined nature of the phase index in the presence of phase defects. The extended approach, by computing the phase index around rather than through phase defects, restores theoretical consistency: the index theorem is satisfied in all cases, and the detected singularities carry genuine interpretive value as markers of rotational activity."

Beyond theoretical consistency, the extended approach offers a conceptually distinct alternative to conventional false-positive suppression. Existing post-processing strategies such as phase singularity clustering or minimum-duration filters implicitly assume phase continuity and rely on heuristic parameters that lack direct physiological interpretation. The extended approach instead grounds false-positive suppression in the mathematics of the phase index: by explicitly modeling phase discontinuities, every detection corresponds to a complete or near-complete rotation around a phase defect, eliminating the need for arbitrary post-processing. Additionally, the extended approach also avoids the false negatives that the naive approach introduces near regions of undefined phase, such as scar tissue or anatomical boundaries. While the phase-difference threshold used here provides a practical means of identifying discontinuities, more sophisticated characterizations of phase continuity remain an avenue for future work."

17. *The authors do not acknowledge recent deep learning techniques for phase mapping and singularity detection: - Mulimani et al., Deep-learning-assisted detection and termination of spiral and broken-spiral waves in mathematical models for cardiac tissue, Phys. Rev. Res., 2020 - Lebert et al., Rotor localization and phase mapping of cardiac excitation waves using deep neural networks, Front. Phys., 2021 - Deng et al., Smart optogenetics for real-time automated control of cardiac electrical activity. Advanced Science, 2026 In particular, these techniques could be trained to detect phase defects and other related structures, and it would be interesting to discuss such applications under future work.*

    answer: We have added these works in the introduction.

    "Together, these features underscore the need to reconsider how singularity detection algorithms handle such factors and previous work has already explored deep-learning techniques to overcome these features [12, 9, 4]. This paper argues that reliable detection is achievable by using phase mapping, without resorting to alternative approaches."

    However, we still consider these works as an alternative approach to the conventional naive phase mapping. Additionally, we mentioned other works that could be useful to detect phase defect which can be use as input for phase mapping.

    "Furthermore, methods that leverage machine learning could also be useful to identify phase defects as an input to phase mapping, without replacing the phase mapping analysis itself [14, 7]."

18. *Line 33: The authors write "... restore the reliability of phase mapping". Shouldn't it be phase singularity / defect tracking? The method to compute the phase is a conventional one.*

    answer: We have clarified the distinction in the introduction:

    "Combined, these results show that naive implementations fail when confronted with discontinuous or unexcitable regions in the phase map, but that reliability can be restored when phase defects are treated properly."

19. *In line 261, the section number is missing.*

    answer: We thank the reviewer for noticing. This issue has been fixed.

    "To illustrate the key features of each simulation, results are shown for a representative single time frame. The full analysis code and videos are provided as supplementary, (see S1 Video and S1 Code), enabling inspection of other time frames."

S1 Video. Supporting videos. All supplementary videos can be found on Zenodo (`http://doi.org/10.5281/zenodo.17602155`).

References

[1] Sergio Alonso, Markus Bär, and Blas Echebarria. Nonlinear physics of electrical wave propagation in the heart: a review. *Reports on Progress in Physics*, 79:096601, 9 2016.

[2] Louise Arno, Jan Quan, Nhan T. Nguyen, Maarten Vanmarcke, Elena G. Tolkacheva, and Hans Dierckx. A phase defect framework for the analysis of cardiac arrhythmia patterns. *Frontiers in Physiology*, 12, 9 2021.

[3] Jörn Davidsen, Leon Glass, and Raymond Kapral. Topological constraints on spiral wave dynamics in spherical geometries with inhomogeneous excitability. *Physical Review E*, 70(5), 11 2004.

[4] Shanliang Deng, Niels Harlaar, Juan Zhang, Sven O. Dekker, Nina N. Kudryashova, Huiling Zhou, Cindy I. Bart, TianYi Jin, Georgy Derevyanko, Willem van Driel, Alexander V. Panfilov, René H. Poelma, Antoine A. F. de Vries, GuoQi Zhang, Tim De Coster, and Daniël A. Pijnappels. Smart optogenetics for real-time automated control of cardiac electrical activity. *Advanced Science*, 13, 4 2026.

[5] Mattias Duytschaever, Robin Van den Abeele, Niels Carlier, Arthur Santos Bezerra, Bjorn Verstraeten, Sebastiaan Lootens, Karel Desplenter, Arstanbek Okenov, Timur Nezlobinsky, Dipen Shah, Annika Haas, Armin Luik, Jordi Martens, Milad El Haddad, Maarten De Smet, Benjamin De Becker, Clara Francois, Jean-Benoit Le Polain de Waroux, Rene Tavernier, Sebastien Knecht, Sander Hendrickx, and Nele Vandersickel. Atrial topology for a unified understanding of typical and atypical flutter. *Circulation: Arrhythmia and Electrophysiology*, 17(11), November 2024.

[6] Mattias Duytschaever, Robin Van den Abeele, Niels Carlier, Arthur Santos Bezerra, Bjorn Verstraeten, Sebastiaan Lootens, Karel Desplenter, Arstanbek Okenov, Timur Nezlobinsky, Dipen Shah, Annika Haas, Armin Luik, Jordi Martens, Milad El Haddad, Maarten De Smet, Benjamin De Becker, Clara Francois, Jean-Benoit Le Polain de Waroux, Rene Tavernier, Sebastien Knecht, Sander Hendrickx, and Nele Vandersickel. Atrial topology for a unified understanding of typical and atypical flutter. *Circulation: Arrhythmia and Electrophysiology*, 17(11):665–667, 11 2024.

[7] Thomas Gaskin, Grigorios A Pavliotis, and Mark Girolami. Inferring networks from time series: A neural approach. *PNAS Nexus*, 3, 3 2024.

[8] Zhang Hong, Hu Bambi, Li Bing-Wei, and Duan Yi-Shi. Topological constraints on scroll and spiral waves in excitable media. *Chinese Physics Letters*, 24(6):1618, jun 2007.

[9] Jan Lebert, Namita Ravi, Flavio H. Fenton, and Jan Christoph. Rotor localization and phase mapping of cardiac excitation waves using deep neural networks. *Frontiers in Physiology*, 12, 12 2021.

[10] Xin Li, Tiago P. Almeida, Nawshin Dastagir, Mar ía S. Guillem, João Salinet, Gavin S. Chu, Peter J. Stafford, Fernando S. Schlindwein, and G. André Ng. Standardizing single-frame phase singularity identification algorithms and parameters in phase mapping during human atrial fibrillation. *Frontiers in Physiology*, 11, 7 2020.

[11] Xinyang Li, Caroline H. Roney, Balvinder S. Handa, Rasheda A. Chowdhury, Steven A. Niederer, Nicholas S. Peters, and Fu Siong Ng. Standardised framework for quantitative analysis of fibrillation dynamics. *Scientific Reports*, 9(1), November 2019.

[12] Mahesh Kumar Mulimani, Jaya Kumar Alageshan, and Rahul Pandit. Deep-learning-assisted detection and termination of spiral and broken-spiral waves in mathematical models for cardiac tissue. *Physical Review Research*, 2, 5 2020.

[13] Peter A. Santucci, Ashwin Bhirud, Smit C. Vasaiwala, David J. Wilber, and Alexander Green. Identification of 2 distinct boundaries distinguishes critical from noncritical isthmuses in ablating atypical atrial flutter. *JACC: Clinical Electrophysiology*, 10(2):251–261, 2 2024.

[14] Christopher Schilling, Matthias Keller, Daniel Scherr, Tobias Oesterlein, Michel Haïssaguerre, Claus Schmitt, Olaf Dössel, and Armin Luik. Fuzzy decision tree to classify complex fractionated atrial electrograms. *Biomedical Engineering / Biomedizinische Technik*, 60, 1 2015.

[15] Masateru Takigawa, Nicolas Derval, Claire A. Martin, Konstantinos Vlachos, Arnaud Denis, Takeshi Kitamura, Ghassen Cheniti, Felix Bourier, Anna Lam, Ruairidh Martin, Antonio Frontera, Nathaniel Thompson, Grégoire Massoulli é, Michael Wolf, Josselin Duchateau, Nicolas Klotz, Thomas Pambrun, Frederic Sacher, Hubert Cochet, Mélèze Hocini, Michel Haï ssaguerre, and Pierre Jaïs. A simple mechanism underlying the behavior of reentrant atrial tachycardia during ablation. *Heart Rhythm*, 16(4):553–561, 4 2019.

[16] Naoki Tomii, Masatoshi Yamazaki, Takashi Ashihara, Kazuo Nakazawa, Nitaro Shibata, Haruo Honjo, and Ichiro Sakuma. Spatial phase discontinuity at the center of moving cardiac spiral waves. *Computers in Biology and Medicine*, 130:104217, 3 2021.

[17] Robin Van Den Abeele, Sebastiaan Lootens, Bjorn Verstraeten, Arthur Santos Bezerra, Arstanbek Okenov, Timur Nezlobinskii, Viktor Van Nieuwenhuize, Sander Hendrickx, and Nele Vandersickel. Paired reentries maintain ventricular tachycardia: a topological analysis of arrhythmic mechanisms using the index theorem. *Frontiers in Network Physiology*, 5:1638085, 2025.

[18] L. Wieser, M. C. Stühlinger, F. Hintringer, B. Tilg, G. Fischer, and L. J. Rantner. Detection of phase singularities in triangular meshes. *Methods of Information in Medicine*, 46:646–654, 1 2007.

[19] Arthur T. Winfree. Varieties of spiral wave behavior: An experimentalist's approach to the theory of excitable media. *Chaos: An Interdisciplinary Journal of Nonlinear Science*, 1:303–334, 10 1991.
