# Supplementary material for: Going around phase defects: Addressing a fundamental flaw of phase singularity detection
Source: PLoS Comput Biol. 2026 Oct 13;22(10):e1013963. doi: 10.1371/journal.pcbi.1013963 (PMC13705599; doi:10.1371/journal.pcbi.1013963)
Supplement: Supplementary file 3 [file pcbi.1013963.s006.pdf]

EPM paper review: second revision

2026-07-30

We thank the reviewers for their careful reading of this manuscript and for their valuable comments. We hope that we have addressed all points raised and believe that the paper has been significantly improved thanks to their constructive feedback.

1 REVIEWER 2

1. *The description of the "optimal" threshold being the minimum to remove defects should be in the Methods not Limitations, same paragraph as the other information about the threshold.*

   answer: We thank the reviewer for this helpful and correct comment. We have removed the description from Limitations and updated the corresponding paragraph in Materials and Methods as follows:

   The thresholds for simulated, experimental, and clinical data were hand-selected by visual inspection of the activation maps. The corresponding values were 0.1π*,* 0.4π*, and* 0.2π*, respectively. These values were selected to ensure accurate annotation of regions where conduction block was expected. The "optimal" threshold would be the lowest value for which only the phase defect is removed. Nevertheless, low threshold values should be used with caution, as they can lead to slow-conducting regions being misclassified as conduction blocks due to the high local phase gradient. In addition, mesh faces containing at least one node with an undefined phase are also removed.*

2. *There is some interpretation in the Results e.g "which is poorly interpretable and far from realistic" and "suggesting that the naive approach does not result in the correct diagnosis". When the Results and Discussion are separate, these comments should be in the Discussion. Results is for what happened, Discussion is for interpretation and contextualisation.*

   answer: We also completely agree with this comment. We have removed the following phrases from Results:

   - *which is poorly interpretable and far from realistic expectations*
   - *suggesting that the naive approach does not result in the correct diagnosis.*
   - *This indicates that more complex activation patterns are present than previously described in the simulations, where the phase index was always equal to minus one, zero, or one.*

   As requested previously, we have kept the Discussion succinct and have not brought back the case-specific phrases that were removed.

3. *Be precise and quantitative throughout the Results section. Instead of "multiple" phase singularities around the defect per time frame (Parallel activation around a functional conduction block),*

   - *give me an average, or a rate.*

     answer: we have changed our wording to be more precise:

     - linear core: *on average two positive and three negative phase singularities*
     - parallel activation: *an average of six positive and six negative phase singularities*

- *Are these values typical, or if not, is there a reason they are bigger/smaller?*

  answer: If "typical" means relative to other simulations: we don't have a large enough sample to state this rigorously. Our expectation is that the number of detections scales with the complexity of the input simulation, so a single "typical" value may not be meaningful across different cases.

  If "typical" means relative to other detection methods: we haven't run a direct comparison, but we'd expect the naive method to over-detect, since it includes no postprocessing step to filter false positives.

- *Similarly, you say the naive approach "generally" detects zero since phase within scar is undefined (Complete and near-complete rotation around scar tissue), but there is a deviation from 0 in the graph in Fig 9. Is it a false positive? Why is it there?*

  answer: We added the following phrase to explain the deviations from zero in Fig 9: *Occasionally, tissue adjacent to the scar briefly remains refractory, forming a small functional block attached to the structural one. The naive approach can then detect a phase singularity at this location.*

- *Finally, you say "several" static non-critical phase defects are consistently annotated (CARTO map of clinical atrial tachycardia) - how many?*

  answer: Also here we have changed our wording to be more precise:

  35 static non-critical phase defects
