# Supplementary material for: Going around phase defects: Addressing a fundamental flaw of phase singularity detection
Source: PLoS Comput Biol. 2026 Oct 13;22(10):e1013963. doi: 10.1371/journal.pcbi.1013963 (PMC13705599; doi:10.1371/journal.pcbi.1013963)
Supplement: Peer review history

# Peer review: Decision Letter 0

**DOI:** 10.1371/journal.pcbi.1013963.r001

**Authors:** Mark Alber, Emma Lejeune

22 Apr 2026

Going around phase defects: reliable phase mapping for realistic data

PLOS Computational Biology

Dear Dr. Verstraeten,

Thank you for submitting your manuscript to PLOS Computational Biology. After careful consideration, we feel that it has merit but does not fully meet PLOS Computational Biology's publication criteria as it currently stands. Therefore, we invite you to submit a revised version of the manuscript that addresses the points raised during the review process.

Please submit your revised manuscript by Jun 22 2026 11:59PM. If you will need more time than this to complete your revisions, please reply to this message or contact the journal office at ploscompbiol@plos.org. When you're ready to submit your revision, log on to https://www.editorialmanager.com/pcompbiol/ and select the 'Submissions Needing Revision' folder to locate your manuscript file.

Please include the following items when submitting your revised manuscript:

* A letter that responds to each point raised by the editor and reviewer(s). You should upload this letter as a separate file labeled 'Response to Reviewers'. This file does not need to include responses to formatting updates and technical items listed in the 'Journal Requirements' section below.

* A marked-up copy of your manuscript that highlights changes made to the original version. You should upload this as a separate file labeled 'Revised Manuscript with Track Changes'.

* An unmarked version of your revised paper without tracked changes. You should upload this as a separate file labeled 'Manuscript'.

If you would like to make changes to your financial disclosure, competing interests statement, or data availability statement, please make these updates within the submission form at the time of resubmission. Guidelines for resubmitting your figure files are available below the reviewer comments at the end of this letter

We look forward to receiving your revised manuscript.

Kind regards,

Emma Lejeune

Academic Editor

PLOS Computational Biology

Mark Alber

Section Editor

PLOS Computational Biology

**Journal Requirements:**

1) Please ensure that the CRediT author contributions listed for every co-author are completed accurately and in full.

At this stage, the following Authors/Authors require contributions: Fu Siong Ng. Please ensure that the full contributions of each author are acknowledged in the "Add/Edit/Remove Authors" section of our submission form.

The list of CRediT author contributions may be found here: https://journals.plos.org/ploscompbiol/s/authorship#loc-author-contributions

2) We ask that a manuscript source file is provided at Revision. Please upload your manuscript file as a .doc, .docx, .rtf or .tex. If you are providing a .tex file, please upload it under the item type u2018LaTeX Source Fileu2019 and leave your .pdf version as the item type u2018Manuscriptu2019.

3) Please upload all main figures as separate Figure files in .tif or .eps format. For more information about how to convert and format your figure files please see our guidelines:

https://journals.plos.org/ploscompbiol/s/figures

**Reviewers' comments:**

Reviewer's Responses to Questions

**Comments to the Authors:**

**Please note here if the review is uploaded as an attachment.**

Reviewer #1: The authors present a very nice method for dealing with phase defects which can

cause spurious detection of phase singularities which is an important

consideration when using phase analysis. The authors show simulations,

experimental and clinical data to illustrate how their method may be used. The

methods are sound and the results convincing. The paper is well written and

organized.

The reviewer's only major question is how valid is Eqn 5 when interpreting 3D

data on a surface. This does not detract from its usefulness in 2D. In 3D,

phase singularities can be I, U, or O-shaped filaments which may or may not

have end points on the measured surface. This is particularly pertinent for

ventricles, but may also be an issue with left atrial with high endo- and epi

dissociation. Does the theory hold that charge is neutral for manifolds sampled

from the 3D space? How would scroll waves in the septum affect interpretation

on the epicardium. In the example given, the artificial boundary cuts out the

septal influence so this is not an issue, but if the heart were rotated 90

degrees about the long axis, the septum wold be in the center of view.

Figure 2: a scale bar or indication of size would be appreciated.

line 261: missing section

line 266: finitewave should be 2 words

Reviewer #2: The review comments have been uploaded as an attached PDF.

Reviewer #3: In their paper “Going around phase defects: reliable phase mapping for realistic data,” by Bjorn Verstraeten et al., the authors introduce an extension of the popular concept of a phase singularity, commonly used in cardiac electrophysiology studies to characterize ventricular fibrillation. The authors identify limitations associated with the “naive” approach and present a few convincing cases in which their extension provides more consistent and precise results. In particular, the method appears to be very well suited for the case of near-complete rotations around larger obstacles, a situation which may arise in atrial tachycardia. While the method is very convincing in this case, its claimed benefit for ventricular fibrillation is unclear. The study provides an incremental but valuable improvement to a well-established method. Beyond the method, the study does not offer new insights into cardiac arrhythmia mechanisms or the nature of spiral wave dynamics.

My main concern is that the method is demonstrated on only a few datasets. Especially, the primary potential use case, clinical data of atrial tachycardia or data with multiple near-complete rotations, is shown for only one case and should be explored more carefully using other examples. In particular, I am curious how noise affects the analysis.

With ventricular fibrillation data, the main improvement seems to be simply avoiding false-positive detections. However, as the authors note, such false-positive detections can also be removed using previously published techniques. Considering this, what is the added benefit of the method for ventricular fibrillation? Is there something new we can learn about ventricular fibrillation with this method?

In the conclusion, the authors write: “These findings challenge the classical view that rotors or functional reentry in cardiac arrhythmia are sustained by physical rotation around singular points.” The term “challenge” is not appropriate in my view as the authors do not fully demonstrate their technique with 2D observations of scroll waves and appear to miss the point that the concept of a phase singularity was not invented to characterize rotor dynamics in detail, but rather to simplify the complexity of wave patterns measured across the surface of the ventricles during ventricular fibrillation. While the proposed extensions can be useful in some instances, other researchers may not necessarily want to adopt these concepts for their data, as a singular point is easier to track and visualize over time than lines and loops.

Numerous studies have tracked rotor cores and characterized drift and rotor core trajectories using phase singularities. Different spiral wave regimes and model parameters were associated with different meandering patterns. Such patterns take the shape of an epitrochoid or hypotrochoid with inward or outward petals. The present study is lacking such an analysis. The authors should either add a paragraph discussing how their framework would impact such studies and forms of analysis, or present new data characterizing meandering rotors while providing a succinct and meaningful analysis of such patterns that provides new insights into spiral wave dynamics.

The study does not account for 3D dynamics. If the authors aim to make a meaningful contribution to the analysis of ventricular fibrillation, they should demonstrate their technique with a 2D/3D analysis by comparing the wave patterns on the surface versus within the volume.

The main finding of the paper, in my view, is shown in Fig. 10, in which the phase defects are displayed as lines that surround the large holes, while short-lived phase singularities are ignored. This figure needs more details. A few other views and other snapshots of different phases of the recording would be helpful to better assess the performance. The caption should detail what the red and blue lines mean physiologically. For example, do they correspond to a clockwise or counter-clockwise rotating activation?

Fig. 6 would benefit from additional snapshots showing the rotor over several time steps. Please create additional subpanels, which show the rotor pattern at different time steps as in the corresponding video. Otherwise, statements in the text, such as “The naive approach identifies one or multiple phase singularities of both positive and negative phase index values at the central defect per time frame.” (line 270ff) do not match with the figure. Moreover, Fig. 6 would benefit from annotations, e.g. arrows pointing at the different components, index values, etc.. Also, the phase singularities and defect lines could be drawn a bit thicker.

In Fig. 6, why are there sometimes two blue lines at the rotor core and what do they mean?

In Fig. 6, what meaning does the outer boundary phase defect line have? What practical relevance does it have? In the discussion the authors write that “… the linear rotor core and a single static critical phase defect denoting the outer edge of the tissue. This is interpreted as paired reentry, with a central semi-stable complete rotation and a stable near-complete rotation at the boundary.” What is the physiological relevance of this sentence? Please clarify.

In Fig. 7, please indicate the propagation direction with an arrow.

It is not clear to me what the added value of the new method is when applied to ventricular fibrillation. In Fig. 9, it appears that the only difference is that the phase singularities are replaced by little rings and that short-lived phase singularities disappear. For the case of ventricular fibrillation, what can this method visualize that the naive method cannot?

Do the little rings in the ventricular fibrillation dataset correspond to micro-anatomical obstacles?

In lines 285-286, it is stated that “the extended approach finds an average of zero critical phase defects and seven non-critical phase defects per time frame”. This is not obvious from the figure. Please modify the figure so that individual phase defect detections can be seen over time. What do these numbers reveal?

In Fig. 10, the LAT and phase maps are discontinuous. Are they supposed to be like that? If not, then this should be improved and be displayed without discontinuities. Also, please add annotations of the anatomical features and defects.

The supplementary videos are not referenced correctly in the text.

In line 261, the section number is missing.

The sentence at the beginning of the discussion, “This section interprets the results presented above, …“, is redundant. The whole section could be more succinct.

Line 33: The authors write “… restore the reliability of phase mapping”. Shouldn’t it be phase singularity / defect tracking? The method to compute the phase is a conventional one.

The authors do not acknowledge recent deep learning techniques for phase mapping and singularity detection:

- Mulimani et al., Deep-learning-assisted detection and termination of spiral and broken-spiral waves in mathematical models for cardiac tissue, Phys. Rev. Res., 2020

- Lebert et al., Rotor localization and phase mapping of cardiac excitation waves using deep neural networks, Front. Phys., 2021

- Deng et al., Smart optogenetics for real-time automated control of cardiac electrical activity. Advanced Science, 2026

In particular, these techniques could be trained to detect phase defects and other related structures, and it would be interesting to discuss such applications under future work.

**********

**Have the authors made all data and (if applicable) computational code underlying the findings in their manuscript fully available?**

The PLOS Data policy requires authors to make all data and code underlying the findings described in their manuscript fully available without restriction, with rare exception (please refer to the Data Availability Statement in the manuscript PDF file). The data and code should be provided as part of the manuscript or its supporting information, or deposited to a public repository. For example, in addition to summary statistics, the data points behind means, medians and variance measures should be available. If there are restrictions on publicly sharing data or code —e.g. participant privacy or use of data from a third party—those must be specified.

Reviewer #1: Yes

Reviewer #2: Yes

Reviewer #3: Yes

**********

PLOS authors have the option to publish the peer review history of their article (what does this mean?). If published, this will include your full peer review and any attached files.

If you choose “no”, your identity will remain anonymous but your review may still be made public.

**Do you want your identity to be public for this peer review?**  For information about this choice, including consent withdrawal, please see our Privacy Policy.

Reviewer #1: No

Reviewer #2: No

Reviewer #3: No

[NOTE: If reviewer comments were submitted as an attachment file, they will be attached to this email and accessible via the submission site. Please log into your account, locate the manuscript record, and check for the action link "View Attachments". If this link does not appear, there are no attachment files.]

**Figure resubmission:**

**Reproducibility:**

?>

Attachment Submitted filename: Round 1 Revisions.pdf

# Author comment: Author response to Decision Letter 1

**DOI:** 10.1371/journal.pcbi.1013963.r002

7 Jul 2026

Attachment Submitted filename: Response to Reviewers.pdf

# Peer review: Decision Letter 1

**DOI:** 10.1371/journal.pcbi.1013963.r003

**Authors:** Stacey Finley, Emma Lejeune

23 Jul 2026

PCOMPBIOL-D-26-00233R1

Going around phase defects: addressing a fundamental flaw of phase singularity detection

PLOS Computational Biology

Dear Dr. Verstraeten,

Thank you for submitting your manuscript to PLOS Computational Biology. After careful consideration, we feel that it has merit but does not fully meet PLOS Computational Biology's publication criteria as it currently stands. Therefore, we invite you to submit a revised version of the manuscript that addresses the points raised during the review process.

Please submit your revised manuscript by Sep 22 2026 11:59PM. If you will need more time than this to complete your revisions, please reply to this message or contact the journal office at ploscompbiol@plos.org. When you're ready to submit your revision, log on to https://www.editorialmanager.com/pcompbiol/ and select the 'Submissions Needing Revision' folder to locate your manuscript file.

Please include the following items when submitting your revised manuscript:

* A letter that responds to each point raised by the editor and reviewer(s). You should upload this letter as a separate file labeled 'Response to Reviewers'. This file does not need to include responses to formatting updates and technical items listed in the 'Journal Requirements' section below.

* A marked-up copy of your manuscript that highlights changes made to the original version. You should upload this as a separate file labeled 'Revised Manuscript with Track Changes'.

* An unmarked version of your revised paper without tracked changes. You should upload this as a separate file labeled 'Manuscript'.

If you would like to make changes to your financial disclosure, competing interests statement, or data availability statement, please make these updates within the submission form at the time of resubmission. Guidelines for resubmitting your figure files are available below the reviewer comments at the end of this letter.

As the corresponding author, your ORCID iD is verified in the submission system and will appear in the published article. PLOS supports the use of ORCID, and we encourage all coauthors to register for an ORCID iD and use it as well. Please encourage your coauthors to verify their ORCID iD within the submission system before final acceptance, as unverified ORCID iDs will not appear in the published article. Only the individual author can complete the verification step; PLOS staff cannot verify ORCID iDs on behalf of authors.

We look forward to receiving your revised manuscript.

Kind regards,

Emma Lejeune

Academic Editor

PLOS Computational Biology

Stacey Finley

Section Editor

PLOS Computational Biology

**Additional Editor Comments (if provided):**

There are a few edits needed to address the remaining reviewer concerns, which will refine and polish the paper.

**Journal Requirements:**

If the reviewer comments include a recommendation to cite specific previously published works, please review and evaluate these publications to determine whether they are relevant and should be cited. There is no requirement to cite these works unless the editor has indicated otherwise.

1) Please amend your detailed Financial Disclosure statement. This is published with the article. It must therefore be completed in full sentences and contain the exact wording you wish to be published.

i) State what role the funders took in the study. If the funders had no role in your study, please state: "The funders had no role in study design, data collection and analysis, decision to publish, or preparation of the manuscript."

ii) If any authors received a salary from any of your funders, please state which authors and which funders..

If you did not receive any funding for this study, please simply state: u201cThe authors received no specific funding for this work.u201d

**Reviewers' comments:**

Reviewer's Responses to Questions

**Comments to the Authors:**

**Please note here if the review is uploaded as an attachment.**

Reviewer #1: All concerns have been addressed.

Reviewer #2: Thank you for responding to my comments; I particularly commend you for your choice of visualisation of naive vs. extended mapping. Much better than stacked bar plots. Please action these minor revisions on the writing.

1. The description of the "optimal" threshold being the minimum to remove defects should be in the Methods not Limitations, same paragraph as the other information about the threshold.

2. There is some interpretation in the Results e.g "which is poorly interpretable and far from realistic" and "suggesting that the naive approach does not result in the correct diagnosis". When the Results and Discussion are separate, these comments should be in the Discussion. Results is for what happened, Discussion is for interpretation and contextualisation.

3. Be precise and quantitative throughout the Results section. Instead of "multiple" phase singularities around the defect per time frame (Parallel activation around a functional conduction block), give me an average, or a rate. Are these values typical, or if not, is there a reason they are bigger/smaller? Similarly, you say the naive approach "generally" detects zero since phase within scar is undefined (Complete and near-complete rotation around scar tissue), but there is a deviation from 0 in the graph in Fig 9. Is it a false positive? Why is it there? Finally, you say "several" static non-critical phase defects are consistently annotated (CARTO map of clinical atrial tachycardia) - how many?

Reviewer #3: I would like to thank the authors for their thorough response. I have no further concerns.

**********

**Have the authors made all data and (if applicable) computational code underlying the findings in their manuscript fully available?**

The PLOS Data policy requires authors to make all data and code underlying the findings described in their manuscript fully available without restriction, with rare exception (please refer to the Data Availability Statement in the manuscript PDF file). The data and code should be provided as part of the manuscript or its supporting information, or deposited to a public repository. For example, in addition to summary statistics, the data points behind means, medians and variance measures should be available. If there are restrictions on publicly sharing data or code —e.g. participant privacy or use of data from a third party—those must be specified.

Reviewer #1: Yes

Reviewer #2: Yes

Reviewer #3: Yes

**********

PLOS authors have the option to publish the peer review history of their article (what does this mean?). If published, this will include your full peer review and any attached files.

If you choose “no”, your identity will remain anonymous but your review may still be made public.

**Do you want your identity to be public for this peer review?**  For information about this choice, including consent withdrawal, please see our Privacy Policy.

Reviewer #1: No

Reviewer #2: No

Reviewer #3: No

[NOTE: If reviewer comments were submitted as an attachment file, they will be attached to this email and accessible via the submission site. Please log into your account, locate the manuscript record, and check for the action link "View Attachments". If this link does not appear, there are no attachment files.]

**Figure resubmission:**

**Reproducibility:**

To enhance the reproducibility of your results, we recommend that authors of applicable studies deposit laboratory protocols in protocols.io, where a protocol can be assigned its own identifier (DOI) such that it can be cited independently in the future. Additionally, PLOS ONE offers an option to publish peer-reviewed clinical study protocols. Read more information on sharing protocols at https://plos.org/protocols?utm_medium=editorial-email&utm_source=authorletters&utm_campaign=protocols

# Author comment: Author response to Decision Letter 2

**DOI:** 10.1371/journal.pcbi.1013963.r004

17 Aug 2026

Attachment Submitted filename: Response to Reviewers - 2.pdf

# Editor report: Decision Letter 2

**DOI:** 10.1371/journal.pcbi.1013963.r005

**Authors:** Stacey Finley, Emma Lejeune

28 Aug 2026

Dear Mr Verstraeten,

We are pleased to inform you that your manuscript 'Going around phase defects: addressing a fundamental flaw of phase singularity detection' has been provisionally accepted for publication in PLOS Computational Biology.

Before your manuscript can be formally accepted you will need to complete some formatting changes, which you will receive in a follow up email. A member of our team will be in touch with a set of requests.

Please note that your manuscript will not be scheduled for publication until you have made the required changes, so a swift response is appreciated.

IMPORTANT: The editorial review process is now complete. PLOS will only permit corrections to spelling, formatting or significant scientific errors from this point onwards. Requests for major changes, or any which affect the scientific understanding of your work, will cause delays to the publication date of your manuscript.

Should you, your institution's press office or the journal office choose to press release your paper, you will automatically be opted out of early publication. We ask that you notify us now if you or your institution is planning to press release the article. All press must be co-ordinated with PLOS.

Thank you again for supporting Open Access publishing; we are looking forward to publishing your work in PLOS Computational Biology.

Best regards,

Emma Lejeune

Academic Editor

PLOS Computational Biology

Stacey Finley

Section Editor

PLOS Computational Biology

***********************************************************

# Editor report: Acceptance letter

**DOI:** 10.1371/journal.pcbi.1013963.r006

**Authors:** Stacey Finley, Emma Lejeune

PCOMPBIOL-D-26-00233R2

Going around phase defects: addressing a fundamental flaw of phase singularity detection

Dear Dr Verstraeten,

I am pleased to inform you that your manuscript has been formally accepted for publication in PLOS Computational Biology. Your manuscript is now with our production department and you will be notified of the publication date in due course.

The corresponding author will soon be receiving a typeset proof for review, to ensure errors have not been introduced during production. Please review the PDF proof of your manuscript carefully, as this is the last chance to correct any errors. Please note that major changes, or those which affect the scientific understanding of the work, will likely cause delays to the publication date of your manuscript.

Soon after your final files are uploaded, unless you have opted out, the early version of your manuscript will be published online. The date of the early version will be your article's publication date. The final article will be published to the same URL, and all versions of the paper will be accessible to readers.

For Research, Software, and Methods articles, you will receive an invoice from PLOS for your publication fee after your manuscript has reached the completed accept phase. If you receive an email requesting payment before acceptance or for any other service, this may be a phishing scheme. Learn how to identify phishing emails and protect your accounts at https://explore.plos.org/phishing.

Thank you again for supporting PLOS Computational Biology and open-access publishing. We are looking forward to publishing your work!

With kind regards,

Anitha Samidurai

PLOS Computational Biology | Carlyle House, Carlyle Road, Cambridge CB4 3DN | United Kingdom ploscompbiol@plos.org | Phone +44 (0) 1223-442824 | ploscompbiol.org | @PLOSCompBiol
